# A novel pan-PI3K inhibitor KTC1101 synergizes with anti-PD-1 therapy by targeting tumor suppression and immune activation

**DOI:** 10.1186/s12943-024-01978-0

**Published:** 2024-03-14

**Authors:** Xin Peng, Xin Huang, Talal Ben Lulu, Wenqing Jia, Shaolu Zhang, Limor Cohen, Shengfan Huang, Jindian Fan, Xi Chen, Shanshan Liu, Yongzhe Wang, Kailin Wang, Sho Isoyama, Shingo Dan, Feng Wang, Zhe Zhang, Moshe Elkabets, Dexin Kong

**Affiliations:** 1 Tianjin Key Laboratory of Technologies Enabling Development of Clinical Therapeutics and Diagnostics, School of Pharmacy, Tianjin Medical University, Tianjin, 300070 China; 2 Key Laboratory of Immune Microenvironment and Diseases (Ministry of Education), Tianjin Medical University, Tianjin, 300070 China; 3 The Shraga Segal Department of Microbiology, Immunology and Genetics, Faculty of Health Sciences, Ben-Gurion University of the Negev, Beer-Sheva, 84105 Israel; 4 Tianjin Medical University Cancer Institute and Hospital, National Clinical Research Center for Cancer, Tianjin’s Clinical Research Center for Cancer, Key Laboratory of Cancer Prevention and Therapy, Tianjin, 300060 China; 5 Tianjin Key Laboratory of Ophthalmology and Visual Science, Tianjin Eye Institute, Tianjin Eye Hospital, Tianjin, 300020 China; 6 State Key Laboratory of Medicinal Chemical Biology, Nankai University, Tianjin, 300071 China; 7 Division of Molecular Pharmacology, Cancer Chemotherapy Center, Japanese Foundation for Cancer Research, Tokyo, 135-8550 Japan; 8 Department of Genetics, School of Basic Medical Sciences, Tianjin Medical University, Tianjin, 300070 China; 9 Department of Pharmacy, Tianjin Medical University General Hospital, Tianjin, 300052 China; 10 International Joint Laboratory of Ocular Diseases (Ministry of Education), Tianjin Medical University, Tianjin, 300070 China

**Keywords:** KTC1101, PI3K inhibitor, Tumor microenvironment, CD8^+^ T cells, Immunotherapy synergy

## Abstract

**Background:**

Phosphoinositide 3-kinases (PI3Ks) are critical regulators of diverse cellular functions and have emerged as promising targets in cancer therapy. Despite significant progress, existing PI3K inhibitors encounter various challenges such as suboptimal bioavailability, potential off-target effects, restricted therapeutic indices, and cancer-acquired resistance. Hence, novel inhibitors that overcome some of these challenges are needed. Here, we describe the characterization of KTC1101, a novel pan-PI3K inhibitor that simultaneously targets tumor cell proliferation and the tumor microenvironment. Our studies demonstrate that KTC1101 significantly increases the anti-PD-1 efficacy in multiple pre-clinical mouse models.

**Methods:**

KTC1101 was synthesized and characterized employing chemical synthesis, molecular modeling, Nuclear Magnetic Resonance (NMR), and mass spectrometry. Its target specificity was confirmed through the kinase assay, JFCR39 COMPARE analysis, and RNA-Seq analysis. Metabolic stability was verified via liver microsome and plasma assays, pharmacokinetics determined by LC–MS/MS, and safety profile established through acute toxicity assays to determine the LD50. The antiproliferative effects of KTC1101 were evaluated in a panel of cancer cell lines and further validated in diverse BALB/c nude mouse xenograft, NSG mouse xenograft and syngeneic mouse models. The KTC1101 treatment effect on the immune response was assessed through comprehensive RNA-Seq, flow cytometry, and immunohistochemistry, with molecular pathways investigated via Western blot, ELISA, and qRT-PCR.

**Results:**

KTC1101 demonstrated strong inhibition of cancer cell growth in vitro and significantly impeded tumor progression in vivo. It effectively modulated the Tumor Microenvironment (TME), characterized by increased infiltration of CD8^+^ T cells and innate immune cells. An intermittent dosing regimen of KTC1101 enhanced these effects. Notably, KTC1101 synergized with anti-PD-1 therapy, significantly boosting antitumor immunity and extending survival in preclinical models.

**Conclusion:**

KTC1101's dual mechanism of action—directly inhibiting tumor cell growth and dynamically enhancing the immune response— represents a significant advancement in cancer treatment strategies. These findings support incorporating KTC1101 into future oncologic regimens to improve the efficacy of immunotherapy combinations.

**Supplementary Information:**

The online version contains supplementary material available at 10.1186/s12943-024-01978-0.

## Introduction

Phosphoinositide 3-kinase (PI3K) plays a pivotal role in linking intracellular and extracellular signal transduction, profoundly influencing cellular processes including growth, proliferation, survival, movement, and metabolism [1, 2]. The PI3K family is primarily divided into three classes (Class I-III), with Class I PI3K comprising four isoforms: PI3Kα, PI3Kβ, PI3Kδ, and PI3Kγ [3, 4]. PI3Kα and PI3Kβ are ubiquitously expressed across various tissues, whereas the PI3Kδ and PI3Kγ isoforms are predominantly found in immune cells [5–7]. Generally, inhibiting PI3Kα and PI3Kβ directly impedes tumor growth, while targeting PI3Kδ and PI3Kγ mainly enhances tumor immunity by modulating immune cell functions [8–13] and shaping the tumor microenvironment (TME) [14, 15]. However, recent studies increasingly highlight the overlapping roles of these four isoforms in both tumor and immune cells [2]. This suggests there is not a one-size-fits-all approach for targeting specific isoforms for desired therapeutic outcomes.

At present, there are five FDA-approved PI3K inhibitors, including Alpelisib (PI3Kα inhibitor), Copanlisib (pan-PI3K inhibitor), Duvelisib (PI3Kδ and PI3Kγ inhibitor), Idelalisib (PI3Kδ inhibitor), and Umbralisib (PI3Kδ inhibitor). Most of these inhibitors have been approved for the treatment of lymphomas, with the exception of Alpelisib, which is uniquely approved for addressing advanced or metastatic breast cancer. Among these, Copanlisib is the only pan-PI3K inhibitor approved. However, its clinical use is confined to intravenous administration and is specifically indicated for adult patients with relapsed or refractory follicular lymphoma (FL) who have undergone at least two prior systemic therapies [16, 17]. This restriction significantly limits its broader clinical application.

Existing PI3K inhibitors often face challenges, including suboptimal bioavailability, potential off-target effects, limited therapeutic indices and cancer-acquired resistance [18, 19]. Approved isoform-selective PI3K inhibitors, claimed to have fewer side effects, have partially mitigated some of these issues [20]. However, the complexity of the PI3K signaling pathway and its extensive feedback mechanisms often triggers the compensatory activation of alternative isoforms upon selective inhibition [9, 21–23]. This compensatory response can result in resistance to PI3K inhibitors. For instance, in vitro treatment with Alpelisib often results in compensatory activation of PI3Kβ [22, 24]. Such feedback loops adaptively readjust existing signaling pathways within the cells, neutralizing the effects of the isoform-selective PI3K inhibitors. To overcome such compensatory mechanisms, utilizing pan-PI3K inhibitors to block all class I PI3K isoforms is a more effective approach. Consequently, there is a growing recognition of the urgent need for next-generation pan-PI3K inhibitors that offer more favorable pharmacological and safety profiles.

Reflecting on the numerous ongoing clinical trials involving PI3K inhibitors, there are over 270 trials, predominantly focusing on cancer treatment (262 trials). These encompass a diverse range of cancers, including 178 targeting solid tumors and 83 for hematological malignancies. Notably, 151 of these trials involve combination therapies with PI3K inhibitors (clinicaltrials.gov, accessed November 2023), highlighting the potential of combinatorial treatment strategies with targeted therapies or immuno-checkpoint blockers.

The advent of immunotherapy, notably through the use of anti-PD-1 antibodies, has revolutionized cancer treatment. These therapies have shown remarkable efficacy in reactivating the immune system to recognize and combat cancer cells. However, the efficacy of such therapies is often compromised by the immunosuppressive nature of the TME [25–27]. Additionally, issues like varying patient responses and the risk of immune-related adverse events (irAEs) further complicate their application [28–31]. To overcome these obstacles, there is a growing trend towards combination therapies. Pairing anti-PD-1 antibodies with other therapeutic agents offers a promising strategy to mitigate the suppressive effects of the TME and amplify the overall efficacy of treatment [32–36].

In this study, we have developed KTC1101, a novel pan-PI3K inhibitor, which emerges as a highly promising drug candidate. It shows potential for superior therapeutic efficacy compared to the currently available pan-PI3K inhibitor Copanlisib and our previously developed clinical trial-phase pan-PI3K inhibitor, ZSTK474 [37, 38]. We thoroughly investigated KTC1101's anti-tumor activity, focusing on its capacity to suppress tumor cell proliferation and stimulate anti-tumor immunity. Moreover, we explored the efficacy of KTC1101 in combination with anti-PD-1 immunotherapy, applying an intermittent dosing schedule aimed at maximizing anti-tumor effects while minimizing adverse side effects. Our findings indicate that KTC1101 is a highly promising PI3K inhibitor and provided a strong rationale to be combined with an immuno-checkpoint blocker, anti-PD-1.

## Methods

### Kinase assay

Procedures for the preparation of KTC1101 are described in supplemental data. To investigate the PI3K inhibitory efficacy of KTC1101, its impact on the kinase activity of PI3K isoforms was assessed using the Adapta kinase assay as previously reported [39]. Initially, a dilution series of 2.5 µL KTC1101 or DMSO (control) was prepared and added to the respective wells of a 384-well plate. Subsequently, optimized 2.5 µL kinase solutions, including PIK3CA/PIK3R1, PIK3CB/PIK3R1, PIK3CG, and PIK3CD/PIK3R1, were introduced to each well. After adding the kinase solutions, 5 µL ATP and PIP2:PS lipid kinase substrate was introduced into each well and incubated for 1 h at room temperature. This step allows for the initiation and progression of the kinase reaction. Following the incubation, a detection solution (5 µL) was added to all the wells. This solution contained 30 mM EDTA to stop the kinase reaction, 6 nM Eu-labeled anti-ADP antibody, and a 3 × concentration of Alexa Fluor® 647 ADP tracer. The EDTA effectively terminates the kinase reaction, while the anti-ADP antibody and ADP tracer are crucial for the detection of ADP produced during the kinase reaction, which correlates with PI3K activity. The plate was then allowed to equilibrate for 30 min at room temperature, ensuring proper binding of the tracer and antibody to the generated ADP. Finally, the fluorescence of each well was measured using a Multi-Mode Microplate Reader (Spark, Tecan). To ascertain the selectivity of KTC1101 for PI3K isoforms and to rule out off-target effects, a kinase panel comprising 50 kinases was tested against KTC1101. This kinase profiling was conducted by Wuhan Heyan Biomedical Technology Co., Ltd.

### Cell lines and culture

The B16 and PC3 cell lines were sourced from the Cell Bank of the Chinese Academy of Sciences (Shanghai, China). The HSC2, HSC4, and CAL33 cell lines were obtained from the American Type Culture Collection (ATCC). S24 cell line was kindly provided by Gloria H. Su of Herbert Irving Comprehensive Cancer Center, Columbia University Irving Medical Center. The TMD8 cell line was generously provided by Dr. Xianhuo Wang of the Tianjin Medical University Cancer Institute and Hospital. Each cell line was maintained in media specified by their respective suppliers, ensuring optimal growth conditions.

### Cell proliferation assay

Cell proliferation was measured by MTT assay as previously described [40]. The cells were seeded in a volume of 200 μL/well in 96-well plates. The next day, cells were treated with DMSO or a series of concentrations of KTC1101. After a 48-h incubation period, 20 μL of MTT (5 mg/mL) was added to each well. After further incubation for 4 h at 37 °C, the absorbance at 490 nm was measured using an iMark microplate reader (BIO-RAD, Hercules, CA, USA).

### Cell cycle analysis

Cell cycle analysis was performed as previously described [41]. Cells were seeded into 6-well plates and treated with DMSO or a series of concentrations of KTC1101. After 48 h of incubation, cells were collected, suspended in PBS, and fixed in 75% ethanol at 4 °C overnight. Next, cells were washed and resuspended in PBS containing PI and RNase. The stained cells were acquired on a BD C6 Flow Cytometer using BD AccuriTM C6 Software (BD Biosciences).

### qRT-PCR

qRT-PCR was performed as previously described [42]. RNA was isolated using Trizol and used to synthesize complementary DNA (cDNA) using a cDNA Synthesis Kit (GenStar), and RT-PCR was performed with aliquots of cDNA samples mixed with SYBR Green MasterMix (Applied Biosystems). Reactions were performed in triplicate. The fold differences in transcripts were calculated using the ΔΔCt method, and 18S rRNA was used as a control to normalize RNA expression (Supplementary Table 1 shows the primers used).

### Western blots

Western blots were performed as previously described [43]. Cells were seeded into six-well plates and treated with DMSO or a series of concentrations of KTC1101. Cells were lysed, and total proteins were harvested. Equal amounts of protein (20–50 μg) were separated using 8% or 12% SDS-PAGE and transferred to polyvinylidene difluoride membranes (Bio-Rad). After blocking in 5% nonfat dry milk, the membranes were incubated with appropriate primary antibodies overnight at 4 °C, washed, and incubated with respective HRP-conjugated secondary antibodies for 1 h at room temperature. The signals were detected using a ChemiDoc XRS + System (Bio-Rad) after exposure to chemiluminescence reagents (Bio-Rad), and β-actin served as the loading control.

### Molecular docking study

For our molecular docking study, we utilized the CDOCKER module of Discovery Studio 3.5 software to predict the binding interactions between PI3K isoforms and KTC1101. This process comprised two primary steps: (1) Preparation of PI3Ks and KTC1101: The initial step involved preparing the PI3K proteins for docking. This was done using the Clean Protein module, which entailed adding hydrogens to the protein, removing any redundant conformations, adding missing residues, and setting the desired pH at 7. Additionally, water molecules within a 5 Å radius around the receptor were deleted to streamline the docking process. The active site of the protein, crucial for the docking simulation, was identified under the “from current selection” section of the receptor-ligand interactions module. The preparation of KTC1101 as the ligand involved assigning it to the Prepare Ligands module. (2) Molecular Docking: The prepared proteins and KTC1101 were then subjected to the docking process using the Dock Ligands (CDOCKER) protocol, providing detailed insights into the binding modes and interactions of KTC1101 with the PI3K isoforms.

### Liver microsome metabolic stability assay

KTC1101 and positive control Midazolam were prepared as 10 mM stock solutions, diluted to 100 µM working solutions in acetonitrile. PBS containing 3 mM MgCl_2_ was prepared by diluting 200 mM MgCl_2_. Microsomes (20 mg/mL) were diluted to 0.56 mg/mL, and a 10 mM NADPH working solution was prepared. 5 µL of the compound working solution was added to 445 µL of microsome working solution in a 96-well plate, pre-incubated at 37°C for 5 min. 50 µL NADPH working solution was added to each well, mixed, and incubated at 37°C on a shaker for 120 min. Samples (40 µL) were collected at 0, 5, 15, 30, 45, 60, and 120 min, added to a 96-well plate, followed by 300 µL acetonitrile containing internal standard (phenacetin). After vortexing for 10 min, samples were centrifuged at 6000 rpm for 10 min. The supernatant (200 µL) was collected for analysis.

### Plasma metabolic stability assay

KTC1101 and positive control Procaine were prepared as 10 mM stock solutions, diluted to 100 µM working solutions in acetonitrile. 495 µL of plasma was placed in a 96-well plate and pre-incubated at 37°C for 5 min. 5 µL of compound working solution was added, mixed, and incubated at 37°C on a shaker for 120 min. Samples (40 µL) were collected at 0, 15, 30, 60, and 120 min, added to a 96-well plate, followed by 300 µL acetonitrile containing internal standard. After vortexing for 10 min and centrifugation at 6000 rpm for 10 min, the supernatant (200 µL) was collected for analysis.

### Pharmacokinetic study of oral administration of KTC1101

Chromatographic conditions employed a Waters BEH C18 column (2.1 mm × 50.0 mm, 1.7 µm) with a mobile phase comprising acetonitrile with 0.1% formic acid (A) and water with 0.1% formic acid (B), at a flow rate of 0.4 mL/min and a column temperature of 40°C. Gradient elution was used: 0–0.3 min, 20:80 → 20:80 (A:B); 0.3–2.5 min, 20:80 → 60:40 (A:B); 2.5–3.0 min, 60:40 → 100:0 (A:B); 3.0–3.01 min, 100:0 → 20:80 (A:B); 3.01–3.5 min, 20:80 → 20:80 (A:B). Mass spectrometry was performed using electrospray ionization (ESI) in positive ion mode, with a capillary voltage of 1 kV, a desolvation temperature of 450 °C, and a desolvation gas flow of 800 L/h. KTC1101 and internal standard phenacetin parameters were optimized for ionization and collision energies.

KTC1101 was prepared at 1 mg/mL concentration in acetonitrile, and further diluted to create a series of standard solutions. Blood samples were taken from BALB/c male mice (19 ± 2g) at various time points post-oral administration of KTC1101 (100 mg/kg). Samples were processed and analyzed using HPLC coupled with mass spectrometry. Data were calculated using Masslynx 4.1 and DAS 3.0 software.

### Acute toxicity study

All mice care and experimental protocols were approved by the Ethical Committee of Tianjin Medical University (permit number: TMUaMEC 2023036). Preliminary testing was conducted on 6 groups of BALB/c mice (half male, half female, 18–22 g), fasted but not water-deprived for 12 h, and administered different doses of KTC1101 (200 to 6400 mg/kg). Post-administration, the animals were observed for 7 days for mortality, body weight changes, and other clinical signs. The LD50 was calculated based on the doses causing 0% and 100% mortality in animals. Subsequent testing was done using doses determined from the preliminary study. Each dose was administered to a single animal, with a 72-h interval between dosages. The administration continued until one of the following criteria was met: (a) three consecutive animals survived; (b) a transition from survival to death occurred in 5 out of 6 consecutive animals; (c) at least four animals were tested after the first transition, with an estimated LD50 range exceeding the critical value (2.5 times). The LD50 and its confidence interval were calculated, and the maximum likelihood method was used to determine the LD50 value using the AOT425StatPgm software.

### Heterotopic nude mouse xenograft model

BALB/c nude, NSG, and C57BL/6J mice were obtained from Tianjin Medical University. The housing temperature was controlled, ranging from 20.5–24 °C, and humidity was monitored but not controlled ranging from 30–70%. The 12-h daily light cycle was from 06:00 to 18:00. To generate a murine subcutaneous tumor model, PC3 and HSC2 cells were subcutaneously injected into the right lateral flank of 4- to 5-week-old male BALB/c nude mice. TMD8, CAL33, and S24 cells were subcutaneously injected into the right lateral flank of 4- to 5-week-old male NSG mice. When tumors reached a volume of 300–500 mm^3^, they were excised, diced into 2 mm × 2 mm × 2 mm pieces, and implanted into the right flanks of BALB/c nude or NSG mice. Tumors were allowed to grow to a volume of 100 mm^3^, and then the animals were randomly divided into several groups. Each group of five mice was treated with either vehicle or KTC1101 (25, 50, or 100 mg/kg, PO). Tumor size was measured every 3 d or 4 d until the endpoint. The tumor volume was calculated using the following formula: length × width × height/2. At the end of the experimental period, mice were euthanized by overdosing on pentobarbital sodium, and the tumors were removed. Tumor tissue was formalin-fixed and paraffin-embedded for histological analysis.

### Syngeneic mouse tumor model

To generate syngeneic mouse models, S24 and B16 cells were subcutaneously injected into the right lateral flank of 4- to 5-week-old male C57BL/6 mice. Tumors were allowed to grow to a volume of 100 mm^3^, and then the animals were randomly divided into several groups. For S24 tumor-bearing mice, each group of five mice was treated with either vehicle or KTC1101 (100 mg/kg, PO). Tumor size was measured every 3 d or 4 d until the endpoint. For B16 tumor-bearing mice, each group of five mice was treated with either vehicle, KTC1101 (50 or 100 mg/kg, PO), anti-PD-1 antibody (250 μg per mouse, clone RPM1-14, Bio X cell, IP), or KTC1101 and anti-PD-1 antibody. Tumor size was monitored every other day, and the tumor was harvested at indicated time points for analysis of tumor-infiltrating lymphocytes. The tumor volume was calculated using the following formula: length × width × height/2.

### Drug concentration measurement in tumors

To evaluate the intratumoral concentration of KTC1101, the B16 murine cancer model was established by subcutaneously implanting 2.5 × 10^5^ B16 cells into the right lateral flank of C57BL/6 mice. The mouse model was utilized for experiments when tumor volumes reached approximately 100 mm^3^. The B16 tumor-bearing C57BL/6 mice were divided into groups, with each group comprising three mice. They were treated with KTC1101 at doses of either 50 or 100 mg/kg (PO). The mice were sacrificed, and tumors were collected on days 1, 5, and 7 post-treatment. Approximately 50 mg of each tumor was homogenized in 1 mL of methanol, followed by centrifugation at 12,000 rpm at 4 °C for 10 min. The concentration of KTC1101 in the supernatant was quantified using LC–MS.

### T cell suppression assay

Spleens from naive mice were isolated and ground through 70-μm filters to generate a single-cell suspension. After RBC lysis, single-cell suspensions were stained with CD3, CD8, CD4, CD25 or CD127 antibodies. Viable CD8^+^ T cells (CD3^+^CD8^+^), CD4^+^ T cells (CD3^+^CD4^+^), and Tregs (CD3^+^CD4^+^CD25^+^CD127^low/−^) from single-cell suspensions were sorted by FACS. Then T cells were labelled with 1 mM CFSE in pre-warmed PBS for 10 min at 37 °C. The CFSE-labelled T cells were seeded in 96-well plates pre-coated with 10 μg/mL CD3 and 1 μg/mL CD28 antibodies and cultured in 1640 media with 10% FBS, 1 mM Sodium Pyruvate (Thermo, 11,360,070), 50 μM β-ME and 100 IU/mL IL2, as well as different concentrations of KTC1101 or solvent control. After 72 h, cells were harvested and the CFSE signal was measured by flow cytometry. Cell growth rate was calculated by the percentage of live, proliferated cell number at each drug concentration vs. solvent control.

### Flow cytometry

To obtain single-cell suspensions for analysis by flow cytometry, tumors were excised, minced, and dissociated in Deoxyribonuclease/collagenase/hyaluronidase buffer (0.1 mg/mL Deoxyribonuclease I, 0.2 mg/mL Collagenase IV and 0.2 mg/mL Hyaluronidase) for 60 min at 37 °C with agitation, and strained through a 70 μm strainer for downstream applications. Tissue was dissociated as described above to obtain single-cell suspensions. Cells were stained with antibodies for 30 min at 4 °C, and then fixed and intracellularly stained using Foxp3/ Transcription Factor Staining Buffer Set (eBioscience) according to the manufacturer’s instructions. Antibodies used in this study can be found in Supplementary Table 2. The stained cells were acquired on a BD C6 Flow Cytometer using BD AccuriTM C6 Software (BD Biosciences) and the data were processed using FlowJo software.

### ELISA detection of secreted cytokines

We examined the secreted cytokines from B16 cells under both in vitro and in vivo conditions. For in vitro-cultured B16 cells, the cells were seeded into six-well plates and treated with either DMSO or a range of KTC1101 concentrations. After 48 h, the total cell count was determined, and the cell culture supernatant was collected for further analysis. For in vivo tumor tissues, the harvested tumor tissues were homogenized. Post-centrifugation, the supernatant was collected and used for protein quantification utilizing a BCA kit. The concentrations of murine CCL5 and CXCL10 in these supernatants were then quantified using specific ELISA kits, in accordance with the manufacturer's instructions.

### H&E and immunohistochemical (IHC) staining

H&E and IHC staining were performed as previously described [44]. H&E staining was used to detect pathological changes in morphology. For histological analysis, formalin-fixed, paraffin-embedded tumors were sectioned, and slides were deparaffinized using xylene (Thermo Fisher). Endogenous peroxidases were quenched with 3% hydrogen peroxide in methanol. Staining was performed using antibodies against phosphorylated Akt (Ser473), phosphorylated S6 (Ser235/236), or Ki67. Counterstaining was performed with Mayer’s hematoxylin (Dako). Slides were observed under an Olympus CX21 microscope and scanned with a high-resolution digital slide scanner (Pannoramic 250, 3DHistech) to capture images.

### Toxicity and safety profiling of KTC1101 in vivo

The B16 tumor-bearing C57BL/6 mice were divided into 4 groups randomly (*n* = 3 per group) and treated with vehicle, KTC1101 (100 mg/kg 3on/4off, PO), anti-PD-1 antibody (250 μg per mouse, IP), or a combination of KTC1101 and anti-PD-1 antibody. After 14 days of treatment, all mice were sacrificed. Blood samples were collected for analysis; one portion was centrifuged (3000 rpm, 4 °C) to obtain serum for biochemical parameter analysis, including alanine aminotransferase (ALT), aspartate aminotransferase (AST), creatinine (CREA), and blood urea nitrogen (BUN). The other portion was used for routine blood examination. Major organs including the heart, liver, spleen, lung, and kidney were collected, sectioned, and stained with H&E for histopathological evaluation.

### JFCR39 COMPARE analysis

JFCR39 drug discovery system [45, 46] was used to measure the growth inhibitory activities and identify the target of KTC1101. The JFCR39 panel consists of 39 cell lines seeded in 96-well plates. After 48-h treatment with KTC1101, cell growth was measured using the sulforhodamine B assay. The concentration of the compound that inhibited 50% of cell growth (GI50) was calculated from the dose–response curve. The deviation of log GI50 for each cell line from the average log GI50 across the JFCR39 panel was plotted as a fingerprint. MG-MID (mean of Log GI50 values) represents the average log GI50 for all 39 cell lines. The most sensitive and least sensitive cell lines were identified by calculating the difference in their log GI50 values (Range) and the difference between the most sensitive cell line and the average GI50 on the JFCR39 panel (Delta). The fingerprint of KTC1101 was compared with the fingerprints of reference compounds in the database. The Pearson correlation coefficient (r) was calculated (*n* = 39). Similarity to a reference compound suggests a similar mode of action or target for KTC1101.

### Transcriptome data analysis

RNA-seq analysis was carried out as previously described [47]. B16 cells were treated with KTC1101 (0.04 and 0.2 μM) or DMSO for 48 h. C57BL/6J mice with subcutaneous tumors of B16 cells were treated with either a vehicle or 100 mg/kg KTC1101 for 3 days, followed by harvesting tumor tissues. Total RNA was isolated using TRIzol reagent. RNA concentrations were quantified using a NanoDrop Spectrophotometer (Thermo Fisher), and RNA integrity was assessed using the RNA Nano 6000 Assay Kit on a Bioanalyzer 2100 system (Agilent Technologies). Samples with RIN values of > 6.0 were used for experiments. A complementary DNA library was prepared, and sequencing was performed according to the Illumina standard protocol by Beijing Novel Bioinformatics Co., Ltd. (https://en.novogene.com/). Specifically, cDNA libraries were prepared using an Illumina NEBNext® UltraTM RNA Library Prep Kit. After cluster generation, the library preparations were sequenced on an Illumina NovaSeq 6000 platform, and 150 bp paired-end reads were generated. For the data analysis, raw data (raw reads) in fastq format were first processed through in-house Perl scripts. Clean reads were obtained by removing reads containing adapters, poly Ns, and low-quality reads from raw data. Reference genome and gene model annotation files were downloaded from the genome website directly. The index of the reference genome was built using Hisat2 v2.0.5, and paired-end clean reads were aligned to the reference genome using Hisat2 v2.0.5. Mapped reads of each sample were assembled using StringTie (v1.3.3b) in a reference-based approach. Feature Counts v1.5.0-p3 was used to quantify the read numbers mapped to each gene. Differential expression analysis was conducted using DESeq2 (v. 1.42.0). Differentially Expressed Genes (DEGs) were visualized as volcano plots using the ggplot2 package (v. 3.4.4). After normalizing FPKM values by Z score, DEGs were selected for hierarchical clustering, displayed as heatmaps, which were generated using the pheatmap package (v.1.0.12). The org.Hs.eg.db package (v.3.17.0) and the org.Mm.eg.db package (v.3.18.0) were used to convert official gene symbol IDs to Entrez IDs, after which DEGs were mapped to KEGG (Kyoto Encyclopedia of Genes and Genomes), GO (Gene Ontology), and Hallmark pathways using the clusterProfiler package (v.4.10.0). Gene Set Enrichment Analysis (GSEA) first calculated the fold change (FC) for each gene between subtypes, then genes were ordered in descending order based on FC values and visualized as volcano plots and ridgeline plots using ggplot2 (v. 3.4.4) and ggrepel (v. 0.9.4) packages. Gene Set Variation Analysis (GSVA) was performed using the GSVA package (v.1.50.0), and results were presented as bubble charts using the ggplot2 package (v. 3.4.4). All analyses were conducted in R v.4.3.1.

### Connectivity map validation

The Connectivity Map (CMap) is a gene expression database developed by the Broad Institute, primarily used to uncover functional connections between small molecule compounds, gene perturbations, and disease states. CMap contains gene expression profiles resulting from various interventions, including small molecule treatments, gene overexpression, and gene knockouts, in different cell lines (PC3, A375, A549, HA1E, HCC515, HEPG2, HT29, MCF7, and VCAP). In this study, we utilized PC3 cells treated with 25 nM KTC1101 for 48 h. Following treatment, RNA-Seq was performed to identify the top 150 upregulated and downregulated differentially expressed genes (DEGs). These DEGs were uploaded to the Connectivity Map (CMap; https://clue.io/) database to analyze the similarity between DEGs and the expression profiles from small molecule drug treatments in the Compound database.

### Tumor immune microenvironment analysis

Cell-type Identification by Estimating Relative Subsets of RNA Transcripts (CIBERSORT) was used to perform a deconvolution analysis of the gene expression matrix based on the principle of linear vector regression. The CIBERSORT algorithm, with a reference to 511 mouse gene signatures, categorized immune cell populations into seven groups and calculated composition scores: B cells including memory, naive, and plasma cells; CD8^+^ T cells including memory, naive, and activated cells; CD4^+^ T cells including memory, naive, follicular, Th1, Th2, and Th17 cells; DCs including activated and immature cells; the rest being mast cells, Tregs, and monocytes.

### Statistical analysis

Data from three independent experiments are presented and expressed as the mean ± SD. An unpaired, 2-tailed Student’s t-test was used for 2-group comparisons. ANOVA with Bonferroni’s correction was used to compare multiple groups. A *p*-value of < 0.05 was considered statistically significant. Before statistical analysis, variations within each group and the assumptions of the tests were checked.

## Results

### Synthesis, characterization and molecular mechanism of KTC1101

**Fig. 1:**
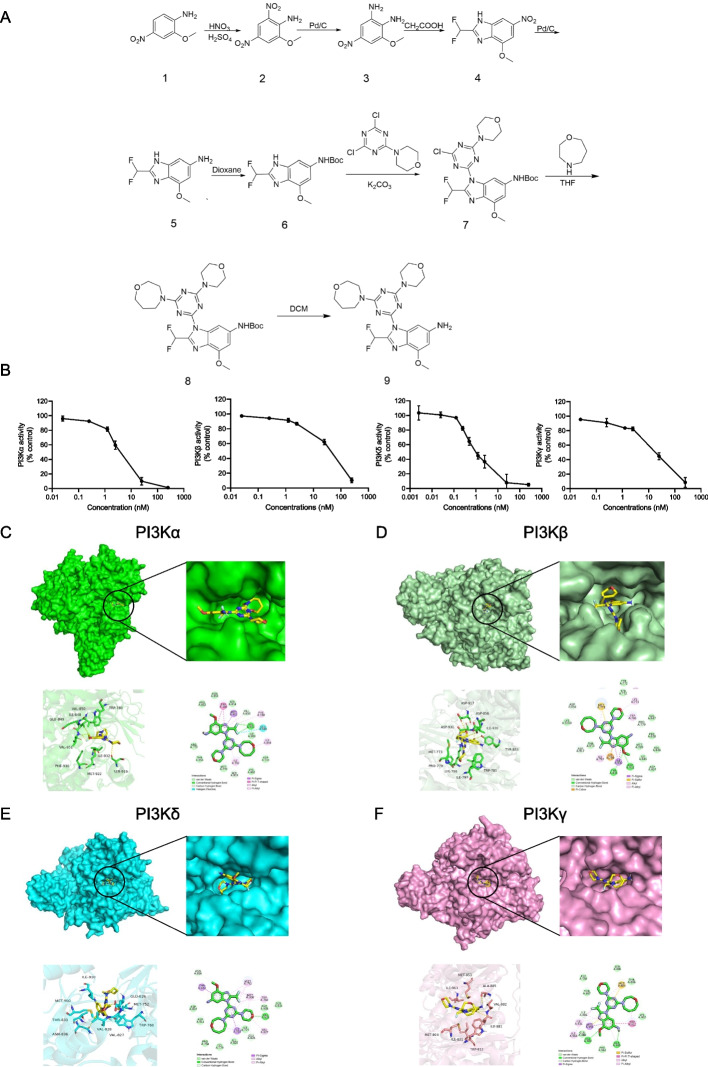
Synthesis and molecular interaction analysis of KTC1101. **A** Synthesis pathway of KTC1101. **B** Evaluation of KTC1101's inhibitory activity on human Class I PI3K isoforms. **C** Analysis of the binding modes between KTC1101 and PI3Kα. **D** Analysis of the binding modes between KTC1101 and PI3Kβ. **E** Analysis of the binding modes between KTC1101 and PI3Kδ. F Analysis of the binding modes between KTC1101 and PI3Kγ

We synthesized KTC1101 through a series of chemical reactions, including nitration and nucleophilic substitution (Fig. 1A). Its molecular structure was confirmed by Nuclear Magnetic Resonance (NMR) spectroscopy and mass spectrometry, ensuring the compound's integrity. High-Performance Liquid Chromatography (HPLC) analysis verified the purity of KTC1101, exceeding 99% (Supplementary Fig. 1A-D). Through Adapta kinase assays, we discovered that KTC1101 exhibited remarkable potency against all PI3K isoforms, as evidenced by its consistently lower IC50 values compared to ZSTK474 [37, 38]. Specifically, KTC1101 exhibits IC50 values of 3.72 nM for PI3Kα, 36.29 nM for PI3Kβ, 1.22 nM for PI3Kδ, and 17.09 nM for PI3Kγ (Fig. 1B). In comparison, ZSTK474 exhibited higher IC50 values across all isoforms: 16 nM for PI3Kα, 44 nM for PI3Kβ, 5 nM for PI3Kδ, and 49 nM for PI3Kγ. This significant improvement in inhibitory efficiency suggests that KTC1101 has stronger potential as a pan-PI3K inhibitor.

To confirm the interaction of KTC1101 with the ATP binding pockets of PI3K isoforms we performed molecular docking. Specifically, in PI3Kα, the benzimidazole group of KTC1101 forms key hydrogen bonds with VAL850 and VAL851, while its morpholine group interacts with SER919. The interaction with the critical hinge amino acid VAL851 notably contributes to its strong inhibition of PI3Kα. Additionally, KTC1101 establishes hydrophobic interactions with TRP780, MET922, PHE930, ILE848, and ILE932, reinforcing its inhibitory mechanism (Fig. 1C). In the case of PI3Kβ, the methoxy group on KTC1101's benzimidazole structure forms hydrogen bonds with TYR833 and ASP931, and its homomorpholine group interacts with ASP856, along with hydrophobic interactions involving LYS771, TRP781, and ILE797 (Fig. 1D). For PI3Kδ, KTC1101's benzimidazole group interacts with ASN836, and its morpholine group with VAL828, a key residue in the binding pocket, suggesting a strong inhibition mechanism. Additional hydrophobic interactions with THR833, MET752, MET900, TRP760, VAL827, and ILE910 are noted (Fig. 1E). In targeting PI3Kγ, KTC1101 forms hydrogen bonds with VAL882 and ALA885, alongside hydrophobic interactions with ILE831, ILE963, MET953, and TRP812 (Fig. 1F). These findings collectively establish KTC1101 as a pan-PI3K inhibitor.

To evaluate the selectivity of KTC1101, we tested its inhibitory activity against a comprehensive panel of 50 kinases. This panel included the four class I PI3K isoforms as well as a broad spectrum of other kinases frequently targeted in therapeutic interventions. Notably, the concentration used for this assessment (1 μM) is significantly higher—by two to three orders of magnitude—than the IC50 for all four PI3K isoforms of KTC1101. At this concentration, KTC1101 robustly inhibited all four PI3K targets with an inhibition rate greater than 90%, while maintaining an inhibition rate of less than 50% for other kinases (Supplementary Fig. 1E), indicating favorable selectivity and remarkable specificity predominantly towards PI3K.

### KTC1101 demonstrates broad-spectrum anti-proliferative activity across diverse tumor cell lines in vitro

**Fig. 2:**
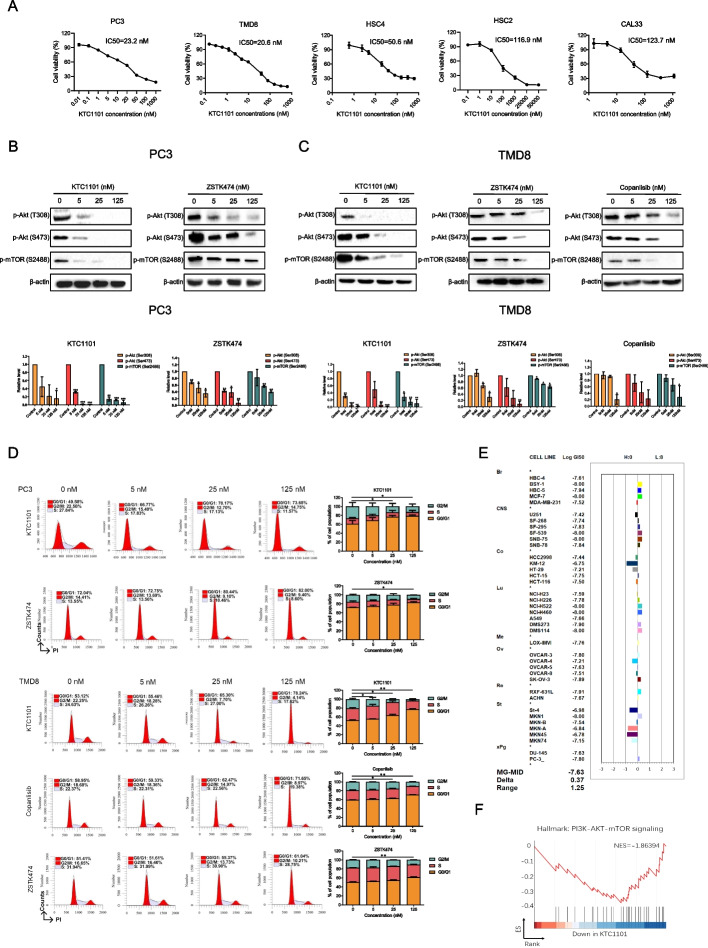
In vitro anti-tumor efficacy of KTC1101.** A** Cell viability assays illustrating KTC1101's anti-proliferative activity against various cancer cell lines after 48-h treatment with incremental concentrations of KTC1101. **B**, **C** The phosphorylation status of Akt and mTOR in PC3 and TMD8 cell lines was analyzed by Western blot after 48-h treatment with increasing concentrations of KTC1101, compared to other pan-PI3K inhibitors ZSTK474 and Copanlisib. **D** Analysis of cell cycle distribution in PC3 and TMD8 cell lines after 48-h treatment with increasing concentrations of KTC1101 or other pan-PI3K inhibitors ZSTK474 and Copanlisib, using propidium iodide (PI) staining. **E** Validation of KTC1101’s inhibitory efficacy across a range of cell lines using the JFCR39 analysis. Cell growth was measured using sulforhodamine B assay. The concentration of KTC1101 that inhibited 50% of cell growth (GI50) was calculated. The deviation of log GI50 for each cell line from the average log GI50 across the JFCR39 panel was plotted as a fingerprint. MG-MID (mean of Log GI50 values) represents the average log GI50 for all 39 cell lines. **F** PC3 cells were treated with 25 nM KTC1101 or vehicle for 48 h. Gene Set Enrichment Analysis (GSEA) depicting alterations in the "PI3K-AKT-mTOR signaling" pathway due to KTC1101 treatment. Graphs are presented as the mean ± SEM from three independent experiments; *p*-values were determined using a two-tailed unpaired Student’s t-test; *p < 0.05; **p < 0.01; ***p < 0.001

To explore the potential anti-tumor activity of KTC1101, we assessed its in vitro anti-proliferative efficacy across a range of tumor cell lines. Cell viability assay demonstrated a dose-dependently anti-tumor activity in prostate cancer PC3 cells, diffuse large B-cell lymphoma TMD8 cells, and head and neck cancer HSC2, HSC4, CAL33 cells, with IC50 ranges between 20nM ~ 130nM (Fig. 2A). Notably, KTC1101 consistently exhibited significantly enhanced inhibitory activity against each of these cell lines compared to Copanlisib and ZSTK474 (Supplementary Fig. 2A). Biochemically, KTC1101 effectively inhibited the PI3K signaling pathway, as evidenced by the reduced phosphorylation of PI3K's downstream effectors, AKT and mTOR. Remarkably, KTC1101 showed more effective inhibition at lower concentrations of AKT and mTOR phosphorylation. This enhanced inhibitory effect was distinctly observed in PC3 cells, compared with ZSTK474. Moreover, KTC1101 also displayed superior inhibitory performance in TMD8 cells, when compared to both Copanlisib and ZSTK474 (Fig. 2B and C, Supplementary Fig. 2B).

We next profiled the response of tumor cells to KTC1101 by measuring cell cycle and apoptosis. KTC1101 treatment induced cell cycle arrest at the G1 phase in all tested cell lines in a dose-dependent manner. Notably, at lower concentrations, KTC1101 exhibits a marginally more pronounced G1 phase blockade compared to the other two inhibitors (Fig. 2D, Supplementary Fig. 2C). This cell cycle inhibition can be explained by the downregulation of cyclin D1 and phosphorylated retinoblastoma protein (p-Rb), along with the upregulation of the cell cycle inhibitor p27 (Supplementary Fig. 2D and E). We did not observe a potent induction of apoptosis except HSC2 cell line (Supplementary Fig. 3A and B). These results align with the typical behavior of PI3K inhibitors [48].

To further validate the broad-spectrum anti-proliferative effects of KTC1101 and establish its corresponding fingerprint profile, we employed the JFCR39 system, comprising 39 human tumor cell lines [39, 46]. KTC1101 exhibited a mean GI50 value of 23.4 nM across all 39 cell lines, significantly lower than that of ZSTK474 (320 nM) and Copanlisib (134 nM), by 13.7-fold and 5.7-fold, respectively (Fig. 2E, Supplementary Fig. 3C). This finding highlights KTC1101's potential as a highly promising pan-PI3K inhibitor, characterized by its broad-spectrum and potent in vitro anti-tumor efficacy. We also observed a substantial correlation (*r* > 0.6) between the effects of KTC1101 and the top-ranked inhibitors (reference compounds) targeting PI3K or its downstream components such as AKT and mTOR (Table 1). This correlation implies that KTC1101 may share a similar antitumor mechanism with these established PI3K/AKT/mTOR inhibitors. Furthermore, we verified the on-target effect of KTC1101 through gene expression profiling analysis, using RNA-Seq data from KTC1101 treated PC3 cells. Our analysis yielded two key points: (i) Comparative analysis with the Connectivity Map (CMap) database revealed that PI3K, AKT, and mTOR inhibitors ranked among the top compounds in the database (Table 2). (ii) Gene set enrichment analysis (GSEA) indicated a reduction in the expression of genes associated with the PI3K/AKT/mTOR pathway in KTC1101-treated cells (Fig. 2F, Supplementary Fig. 3D, Supplementary Tables 3 and 4). This transcriptomic-level evidence further reinforces the characterization of KTC1101 as a potent and typical pan-PI3K inhibitor.

**Table 1:**
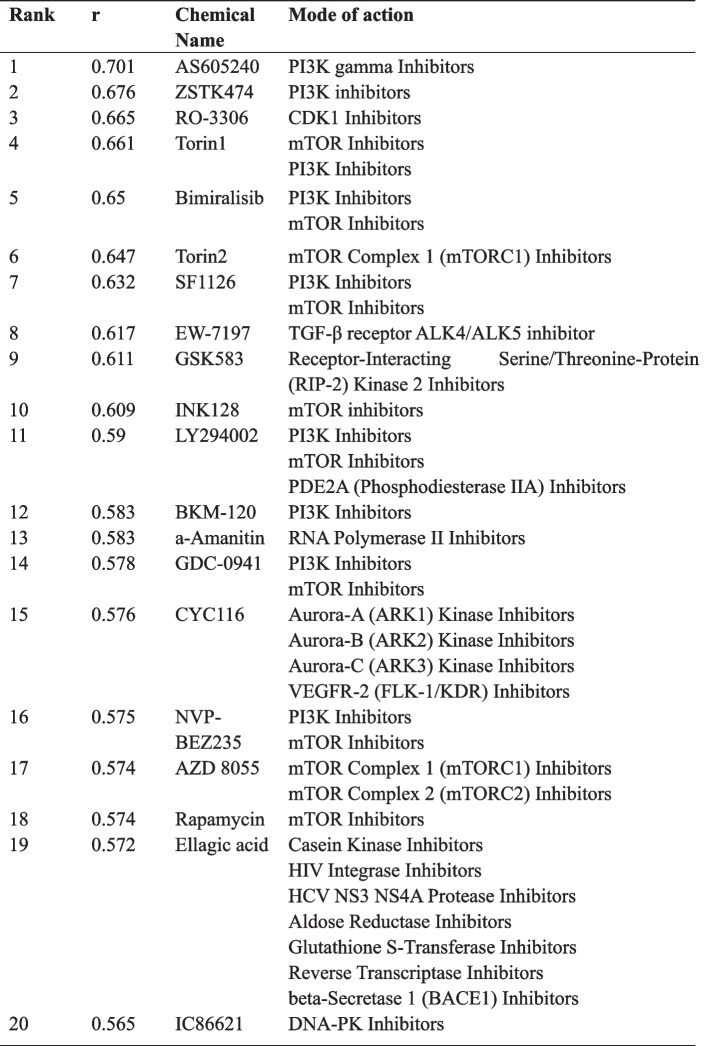
JFCR COMPARE analysis with KTC1101 as a seed

**Table 2:**
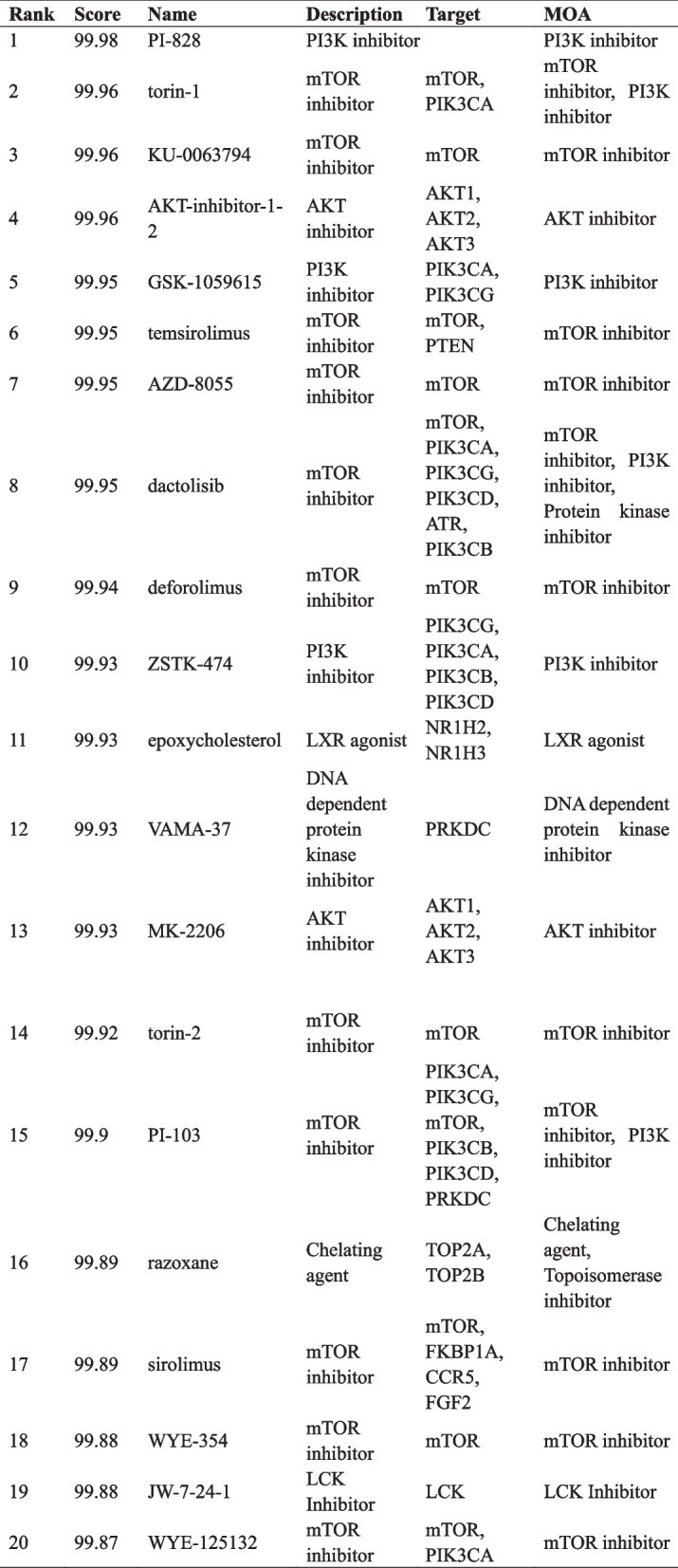
CMap analysis with KTC1101 as a seed

### KTC1101 demonstrates favorable pharmacological properties and pronounced anti-tumor activity in mice

**Fig. 3:**
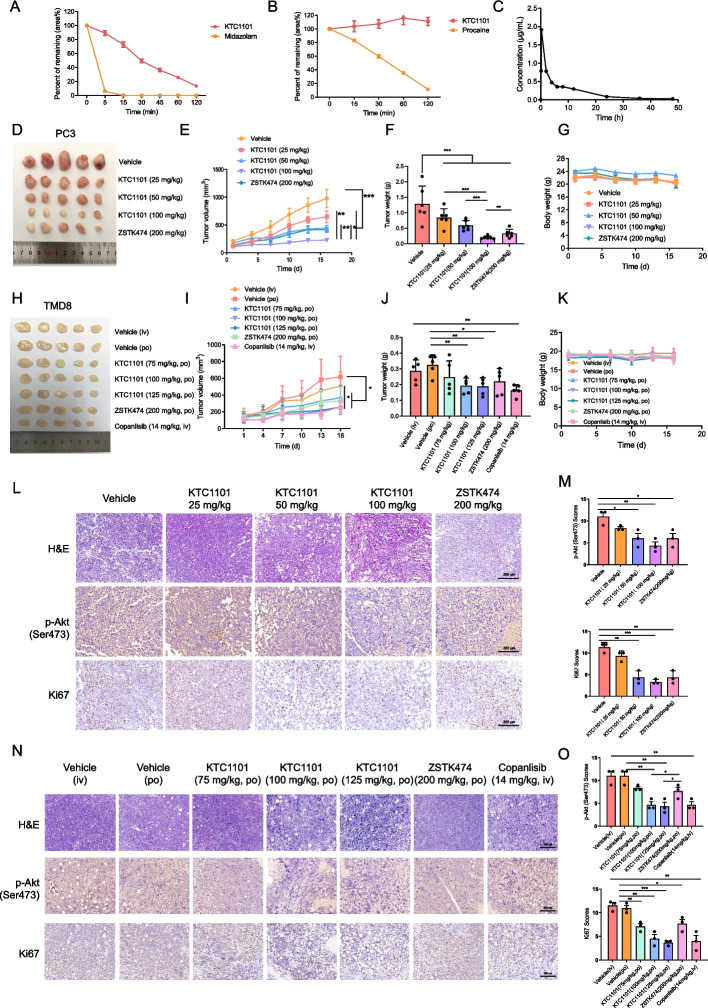
In vivo efficacy of KTC1101 in xenograft models. **A** Graphical representation of KTC1101’s residual percentage (area%) in the mouse liver microsome incubation system. **B** Graph showing the residual percentage (area) of KTC1101 in the mouse plasma incubation system. **C** Blood concentration–time curve following oral administration of KTC1101 (100 mg/kg) in mice. **D** Nude mice with subcutaneous tumors of PC3 cells were treated with either a vehicle or varying doses of KTC1101 (25, 50, or 100 mg/kg, PO) and ZSTK474 (200 mg/kg, PO) (*n* = 5). Tumors are presented post-excision. **E**–**G** Measurements of tumor volumes, tumor weights, and body weights every three days. **H** NSG mice with subcutaneous tumors of TMD8 cells were treated with either a vehicle or varying doses of KTC1101 (75, 100, or 125 mg/kg, PO), ZSTK474 (200 mg/kg, PO), and Copanlisib (14 mg/kg, IV) (*n* = 5). Images of excised tumors are presented. **I**-**K** Tracking of tumor volumes, tumor weights, and body weights at three-day intervals. **L** H&E staining and Immunohistochemistry staining of phosphorylated Akt (p-Akt) and Ki67 in PC3 tumor tissues from mouse xenografts treated with KTC1101 and ZSTK474. Scale bar, 200 μm. **M** Statistical quantification of staining intensities in (**L**), with each point representing the mean of 5 images. **N** H&E staining and Immunohistochemistry staining of phosphorylated Akt (p-Akt) and Ki67 in TMD8 tumor tissues from mouse xenografts treated with KTC1101, ZSTK474 and copanlisib. Scale bar, 200 μm. **O** Statistical quantification of staining intensities in (**N**), with each point representing the mean of 5 images. Graphs are presented as the mean ± SEM from three independent experiments; *p*-values were determined using a two-tailed unpaired Student’s t-test; *p < 0.05; **p < 0.01; ***p < 0.001

We next aimed to characterize the pharmacological properties of KTC1101 in vivo. Specifically, we tested the metabolic stability, oral absorption, and safety profile of KTC1101. We used liver microsome and plasma assays to evaluate metabolic stability and found that KTC1101 exhibited a lower clearance rate compared to the control compound Midazolam, and demonstrated a stable concentration in plasma over 120 min, surpassing Procaine's stability (Fig. 3A and B, Supplementary Tables 5 and 6). The absorption and safety profile of KTC1101 were also assessed in mice. Pharmacokinetic profiling indicated effective oral absorption, characterized by a T_max_ of 0.67 h, a C_max_ of 1.66 μg/mL, and an AUC0-t of 9.33 μg/mL*h (Fig. 3C, Supplementary Table 7). Acute toxicity tests further revealed an LD50 of 2136 mg/kg (Supplementary Table 8). These findings underline the safety profile of KTC1101 and indicate a broad therapeutic window by oral administration.

To evaluate the anti-tumor efficacy of KTC1101 in vivo, we utilized human tumor cell xenograft models in immunodeficient mice. Tumor growth arrest was detected in 100 mg/kg in three out of the four tested tumor models (PC3, CAL33, and HSC2) with no evidence of relapse (Fig. 3D-F, Supplementary Fig. 4A-H). TMD8 xenografts were the only cancer model that slowed tumor progression at 100 mg/kg (Fig. 3H-J). Remarkably, in the PC3 xenograft model, even a lower dose of 25 mg/kg led to a significant delay in tumor growth. The efficacy of 50 mg/kg KTC1101 in inhibiting PC3 tumors was equivalent to 200 mg/kg ZSTK474, and increasing KTC1101's dose to 100 mg/kg further enhanced its effectiveness (Fig. 3D-F), while maintaining safety (Fig. 3G). This indicates KTC1101's superior efficacy against PC3 tumors compared to ZSTK474. In the TMD8 model, oral administration of 100 mg/kg and 125 mg/kg KTC1101 marginally outperformed those of 200 mg/kg ZSTK474 orally and was comparable to 14 mg/kg Copanlisib intravenously (the latter not available for oral administration) (Fig. 3H-J). This indicates KTC1101's potential in anti-lymphoma treatment, especially considering its dosage and oral administration route. There were no statistically significant differences in body weight among the different mouse groups (Fig. 3K). H&E staining revealed no obvious tissue damage (Supplementary Fig. 4I), suggesting minimal toxicity and good tolerability of the drugs. Immunohistochemical analysis of the tumor tissues further corroborated the anti-proliferative effect of KTC1101, indicated by a notable reduction in Ki67 expression, and confirmed its on-target effect with decreased phosphorylation of AKT (Fig. 3L-O). These results collectively indicate that KTC1101 has the potential to surpass the efficacy of existing typical pan-PI3K inhibitors in in vivo studies.

### Intermittent dosing of KTC1101 achieves optimal therapeutic benefits

**Fig. 4:**
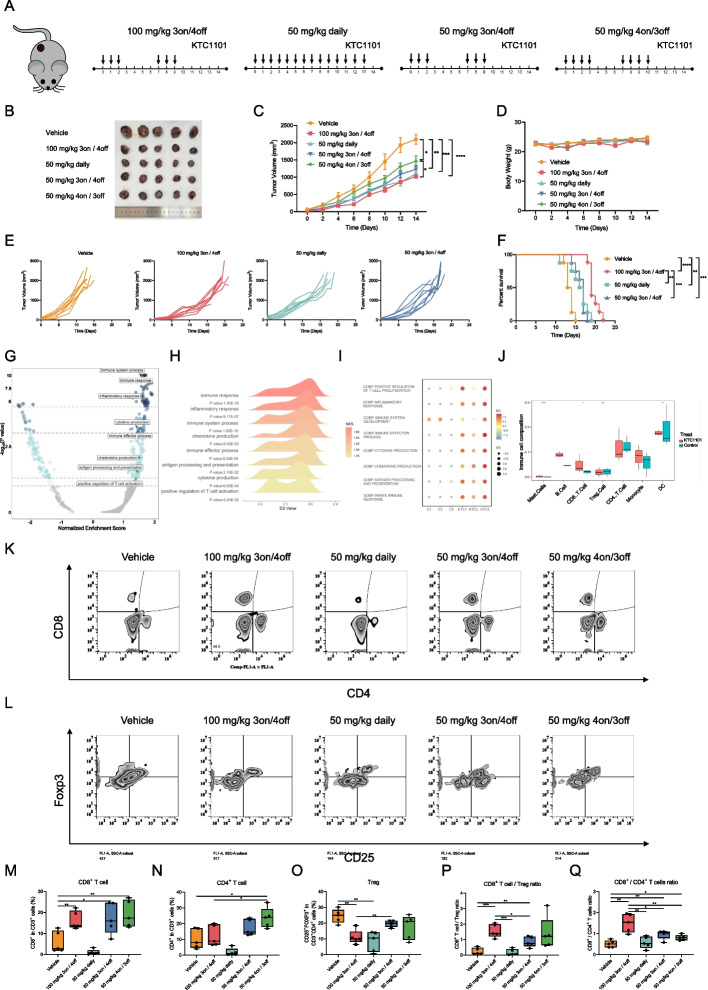
In vivo evaluation of KTC1101's therapeutic efficacy and immune modulation. **A** Schematic representation of treatment regimens applied in thestudy. **B-D** C57BL/6J mice with subcutaneous B16 cell tumors received either vehicle or KTC1101 treatments for 2 weeks (*n* = 5). Post-euthanasia images of the excised tumors (**B**), alongside measurements of tumor volumes (**C**) and body weights (**D**) every two days, are depicted. **E** Individual tumor growth tracking in B16 tumor-bearing mice subjected to KTC1101 or vehicle treatment *(n* = 8). **F** Survival curve analysis of B16 tumor-bearing mice under KTC1101 or vehicle treatment (*n* = 8). **G** C57BL/6J mice with subcutaneous tumors of B16 cells were treated with either a vehicle or KTC1101 for 3 days, followed by RNA-Seq of the tumor tissues. Scatter plot showcasing Gene Set Enrichment Analysis (GSEA) results for differentially expressed genes mapped to Gene Ontology (GO) pathways, including normalized enrichment scores and *p*-values, with horizontal dashed lines indicating *p*-values of 0.1, 0.05, 0.001, and 0.0001. **H** Ridgeline plot visualization of GSEA data highlighting the enrichment of specific pathways in response to KTC1101 treatment, including term names, *p*-values, and enrichment scores, with normalized enrichment scores represented as color variations. **I** Bubble plot displaying the results of Gene Set Variation Analysis (GSVA) for differentially expressed genes, with enrichment scores depicted as color variations and bubble size. **J** Box plot presenting the results of CIBERSORT analysis using 511 mouse gene signatures as a reference, including relative scores of immune cell types and cell population categories. **K** Representative flow cytometric analysis of CD8^+^ and CD4^+^ T cell populations (gated on CD3^+^) in B16 tumors (*n* = 5). **L** Representative flow cytometric analysis of regulatory T cell (Treg cell; CD25^+^Foxp3^+^) cell populations in B16 tumors (*n* = 5). **M** Quantification of CD8^+^ T cell populations in B16 tumors (*n* = 5). **N** Quantification of CD4^+^ T cell populations in B16 tumors (*n* = 5). **O** Quantification of Treg cell populations in B16 tumors (*n* = 5). **P** Quantification of CD8^+^ T cells/Treg ratios in B16 tumors (*n* = 5). **Q** Quantification of CD8^+^/CD4^+^ T cell ratios in B16 tumors (*n* = 5). Graphs are presented as the mean ± SEM from three independent experiments; *p*-values were determined using a two-tailed unpaired Student’s t-test; *p < 0.05; **p < 0.01; ***p < 0.001; ****p < 0.0001

To further profile the anti-tumor activity of KTC1101 and the treatment effect on tumor cells and its microenvironment, we expanded our studies to syngeneic tumor models and investigated the effect on tumor-immune interaction under multiple dosing regimens. Initially, we profiled the response of B16 melanoma cells to KTC1101 in vitro. KTC1101 treatment exhibited a strong anti-proliferative activity against B16 cells, significantly inhibited the PI3K pathway, induced G1 phase arrest, and minimally influenced apoptosis (Supplementary Fig. 5A-F), consistent with observations in other cancer cell lines [48]. Next, in exploring the most effective dosing regimen for KTC1101, we conducted in vivo studies on B16 mouse models, comparing two distinct dosing approaches: intermittent and continuous (Fig. 4A). Our investigation revealed that both high-dose intermittent (100 mg/kg, 3 days on/4 days off) and low-dose continuous (50 mg/kg, daily) administrations, with similar total drug exposures, comparably suppressed tumor growth over 14 days without significant changes in mouse body weight (Fig. 4B-D, Supplementary Fig. 6A). However, the intermittent regimen notably prolonged mouse survival compared to the continuous regimen, suggesting distinct immunological and microenvironmental impacts of these dosing strategies (Fig. 4E and F).

To profile the response in mice, we sought to conduct gene expression profiling and utilized computational tools to estimate the composition of cells in the TME. To this end, RNA-Seq analysis of bulk tumor tissues pre and post-KTC1101 treatment revealed notable shifts in gene expression with 777 genes upregulated and 778 downregulated (Supplementary Fig. 6B and C, Supplementary Table 12). GSEA and GSVA demonstrated enrichment in pathways related to the immune system such as “immune response”, “inflammatory response”, “immune system process”, “chemokine production”, “antigen processing and presentation”, “cytokine production”, “positive regulation of T cell activation” were prominently upregulated at the top across all analyzed pathways (Fig. 4G-I, Supplementary Tables 13 and 14), indicating a strong immune-related response induced by KTC1101 treatment. Furthermore, Cibersort analysis further illuminated an increase in tumor-targeting CD8^+^ T cells and dendritic cells, accompanied by a reduction in immunosuppressive Treg cells following treatment with KTC1101 (Fig. 4J). To confirm these findings, we conducted FACS analysis and demonstrated an increase in intratumoral CD8^+^ T, CD4^+^ T, and a reduction of Treg cells under the various intermittent dosing treatments (Fig. 4K-O). The ratios between CD8^+^ T to Treg and CD8^+^ T to CD4^+^ T indicate that pulsative treatment regimens, characterized by intermittent dosing, are more beneficial compared to chronic, continuous treatment approaches in modulating the TME (Fig. 4P and Q).

These results imply that while continuous dosing is effective in inhibiting tumor cell proliferation, its capability to fully activate tumor immunity may be limited, as evidenced by the suboptimal long-term survival outcomes. This limitation might stem from the accumulation of the drug in continuous dosing (Supplementary Fig. 6D), which could diminish KTC1101's selective advantage in preferentially suppressing Treg cell proliferation over CD8^+^ T cells. Such selectivity is crucial for a potent anti-tumor response, indicating the need for careful consideration of dosing strategies to maximize therapeutic efficacy.

While KTC1101 efficacy seems to be also related to the activation of the immune system, we sought to validate the concept of the findings in an additional syngeneic tumor model and test if anti-tumor immunity machinery is involved in treatment efficacy. To this end, we have used the S24 tumor cells [49], which showed sensitivity to KTC1101 in vitro (Supplementary Fig. 6E and F), and injected them into immunodeficient NSG mice and immunocompetent C57BL/6 mice. When the tumor reached ~ 60mm^3^ mice were treated daily by oral gavage of KTC1101 100 mg/kg, and tumor size was measured twice a week. Comparing the tumor growth kinetics of S24 tumors in WT and NSG mice indicates that in NSG mice tumors started to progress after 10 days, while in WT the relapse was detected after 18 days. This faster progression in NSG mice highlights the contribution of immune responses in its anti-tumor mechanism of KTC1101 (Supplementary Fig. 6G).

### KTC1101 has potential to activate tumor immunity through tumor-cell-intrinsic effects and direct T-cell modulation

**Fig. 5:**
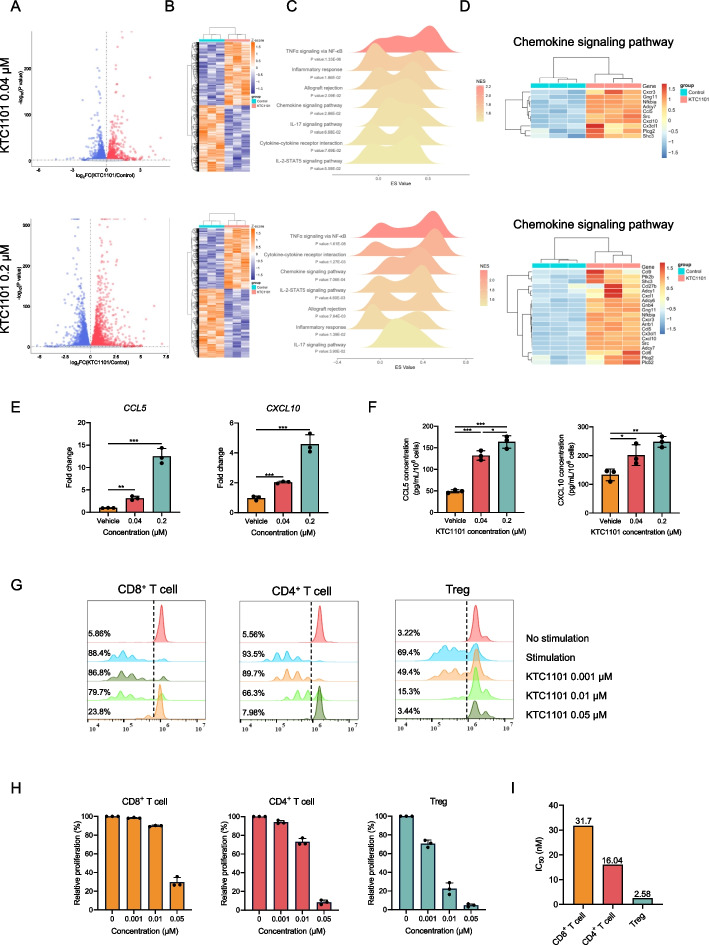
KTC1101's impact on tumor immunity and T-cell response. **A** A volcano plot depicting overall gene expression alterations in B16 cells post 48-h treatment with KTC1101 or vehicle. The plot includes log fold-change values and *p*-values, with a horizontal dashed line signifying a *p*-value threshold 0.05. **B** A heatmap representing the differentially expressed genes. **C** Ridgeline plot illustrating the enrichment of differentially expressed genes in specific KEGG and Hallmark pathways, including term names, *p*-values, and enrichment scores, with normalized enrichment scores represented as color variations. **D** Heatmap of genes in the "Chemokine signaling pathway". **E** An RT-PCR analysis showing the relative expression of crucial chemokines, CCL5 and CXCL10, in B16 cells following a 48-h treatment with KTC1101. **F** ELISA quantification of CCL5 and CXCL10 secretion levels in the supernatant of B16 cell cultures treated with KTC1101 for 48 h. **G** Flow cytometry-based analysis depicting the proliferation of CD8^+^ T cells, CD4^+^ T cells, and Tregs in the presence of KTC1101 for 72 h, with representative histograms and percentage of dividing cell populations. CD8^+^ T cells, CD4^+^ T cells, and Tregs from the spleen of WT mice were sorted by FACS. **H** The differential inhibitory effects of KTC1101 on T cells. **I** IC50 values for KTC1101’s effect on different T cell populations. Graphs are presented as the mean ± SEM from three independent experiments; *p*-values were determined using a two-tailed unpaired Student’s t-test; *p < 0.05; **p < 0.01; ***p < 0.001

To underline the potential mechanism of KTC1101 in CD8^+^ and CD4^+^ T cells infiltration, and reduction of Treg cells, we sought to (i) explore if KTC1101 influences the expression of tumor-derived immune–related factors that regulate T cells infiltration, (ii) explore the direct effect of KTC1101 on the proliferation of T cells subsets. To comprehensively explore whether KTC1101 affects tumor-immune-response signaling in vitro, B16 cells were treated for 48 h with varying concentrations of KTC1101 followed by RNA-Seq. At a lower concentration of KTC1101, 2312 genes were upregulated, and 2375 genes were downregulated; at a higher concentration, 3626 genes were upregulated, and 3754 genes were downregulated (FDR < 0.05, Fig. 5A and B, Supplementary Table 9). GSEA revealed a consistent pattern of pathway activation by KTC1101 across both low and high concentrations. Pathways such as “TNFα signaling via NF-κB”, “inflammatory response”, “chemokine signaling pathway”, “cytokine-cytokine receptor interaction”, and “IL-2-STAT5 signaling pathway” were significantly upregulated at both tested concentrations (Fig. 5C, Supplementary Table 10). This consistent activation across concentrations suggests a strong association between KTC1101 and these crucial immunological pathways. Heatmaps for the most differentially regulated genes in the top GSEA signatures induced by KTC1101 showed increased expression of numerous central pro-inflammatory cytokines and chemokines, including CCL5 and CXCL10, which are crucial for CD8^+^ T lymphocyte chemotaxis [50–52] (Fig. 5D, Supplementary Fig. 7A-C, Supplementary Table 11). These factors secreted in the TME can potentially contribute to an optimal anti-tumor T cell response. The expression levels of CCL5 and CXCL10 upon KTC1101 exposure were significantly stimulated, as confirmed by qRT-PCR and ELISA (Fig. 5E and F). To investigate the potency of KTC1101 on the proliferation of T cell subsets, we sorted CD8^+^ T cells, CD4^+^ T cells, and Tregs and assessed cell proliferation post-KTC1101 treatment. The results indicated that Tregs were most sensitive to KTC1101, followed by CD4^+^ T cells, while CD8^+^ T cells were the least sensitive (Fig. 5G-I). This selective inhibition of Treg proliferation, over a tenfold greater extent than CD8^+^ T cells, underscores KTC1101's potential to shift the immune balance favorably towards an anti-tumor response. Collectively, these findings highlight KTC1101's capability to activate tumor immunity through both intrinsic effects on tumor cells and specific modulation of T cell populations.

### KTC1101 synergizes with anti-PD-1 to enhance anti-tumor effects

**Fig. 6:**
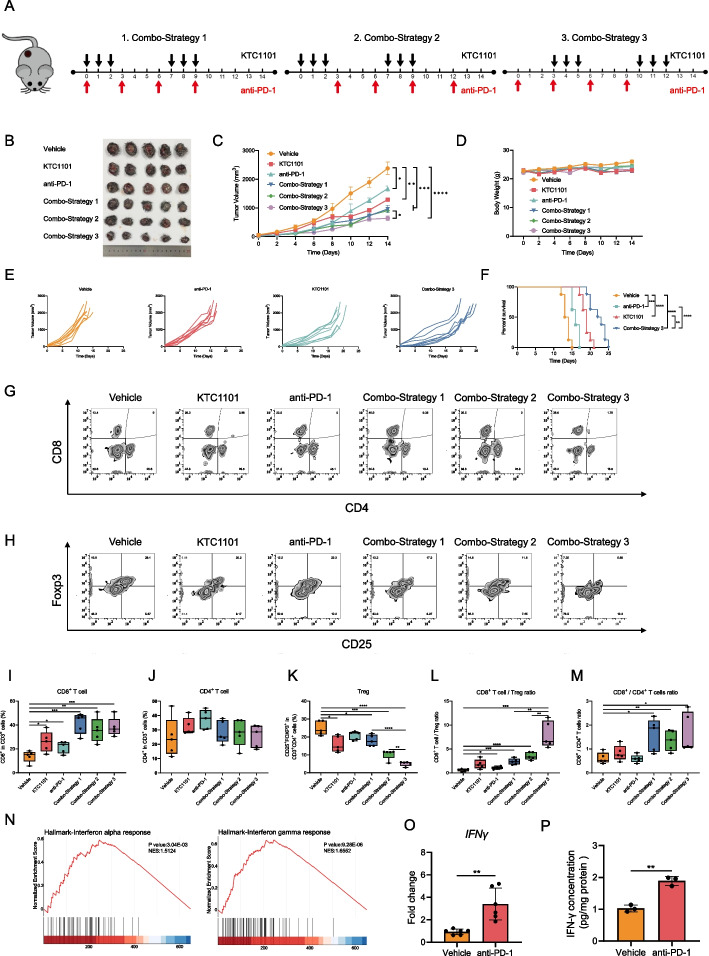
Synergistic effects of KTC1101 and anti-PD-1 therapy in B16 tumor models. **A** Schematic representation of various treatment strategies in B16 mouse models. (**B-D**) C57BL/6J mice with subcutaneous tumors of B16 cells were treated with either a vehicle, KTC1101, anti-PD-1 antibody, or KTC1101 and anti-PD-1 antibody for 2 weeks (*n* = 5). Images of the mice post-euthanasia and the excised tumors are presented (**B**). Tumor volumes (**C**) and body weights (**D**) were measured every two days. **E** Individual tumor growth curves of B16 tumor-bearing mice under different treatment conditions (*n* = 8). **F** Survival analysis of B16 tumor-bearing mice subjected to various treatments (*n* = 8). **G** Flow cytometric analysis of CD8^+^ and CD4^+^ T cell populations (gated on CD3^+^) in B16 tumors (*n* = 5). **H** Flow cytometric analysis of regulatory T cell (Treg cell; CD25^+^Foxp3^+^) populations in B16 tumors (*n* = 5). **I** Quantitative assessment of CD8^+^ T cell populations in B16 tumors (*n* = 5). **J** Quantification of CD4^+^ T cell populations in B16 tumors (*n* = 5). **K** Quantification of Treg cell populations in B16 tumors (*n* = 5). **L** Calculation of CD8^+^ T cells/Treg ratios in B16 tumors (*n* = 5). **M** Quantification of CD8^+^/CD4^+^ T cell ratios in B16 tumors (*n* = 5). **N** C57BL/6J mice with subcutaneous tumors of B16 cells were treated with either a vehicle or anti-PD-1, followed by RNA-Seq of the tumor tissues after 3 days. GSEA plots illustrating changes in “Interferon alpha response,” “Interferon-gamma response” pathways. **O** RT-PCR analysis of IFNγ expression levels in tumor tissues of a group of vehicle or anti-PD-1. **P** ELISA-based quantification of IFNγ secretion levels in tumor supernatants. Graphs are presented as the mean ± SEM from three independent experiments; *p*-values were determined using a two-tailed unpaired Student’s t-test; *p < 0.05; **p < 0.01; ***p < 0.001; ****p < 0.0001

Leveraging the immune-activating properties of intermittent KTC1101, we speculated that supplementation of anti-PD-1 will further improve anti-tumor immunity and will induce a potent anti-tumor activity when combined with KTC1101. To test that hypothesis, we designed three distinct treatment strategies: Strategy 1: entailed simultaneous administration of KTC1101 and anti-PD-1 antibody, Strategy 2: involved KTC1101 pre-treatment followed by combination therapy, and Strategy 3: commenced with anti-PD-1 antibody pre-treatment before combined administration (Fig. 6A). Tumor growth kinetics shows that strategy 3 is the most efficacious approach, significantly suppressing tumor growth and enhancing survival (Fig. 6B-F, Supplementary Fig. 8A). FACS analysis of treated tumors revealed a marked reduction in intratumoral Tregs and an increase in CD8^+^ T cells, enhancing the CD8^+^ T cell/Treg ratio and amplifying anti-tumor immunity in Strategy 3 compared to other strategies (Fig. 6G-M). The enhanced therapeutic outcome observed in Strategy 3 is likely attributed to increased intratumoral IFNγ levels post-PD-1 antibody treatment, rendering Tregs more susceptible to the inhibitory effects of KTC1101 on PI3K [53]. Bulk RNA-Seq and subsequent GSEA reinforced these observations, revealing elevated IFNα and IFNγ responses following anti-PD-1 therapy (Fig. 6N, Supplementary Tables 15 and 16). This was further validated by RT-PCR and ELISA at both the transcriptional and protein levels (Fig. 6O and P). Additionally, Strategy 3 notably promoted dendritic cell infiltration and the transition of macrophages from an M2 to an M1 phenotype, contributing to a more effective anti-tumor immune environment (Supplementary Fig. 8B-G). This combination strategy also exhibited minimal impact on vital organs and primary physiological parameters in mice, indicating its good tolerability (Supplementary Fig. 8H and I, Supplementary Table 17). Collectively, these results suggest that the strategic sequencing of anti-PD-1 inhibition followed by combined administration can effectively transform conventional tumors into immune cell-infiltrated tumors, enhancing the overall anti-tumor response.

### KTC1101's dual-action mechanism in cancer therapy

**Fig. 7:**
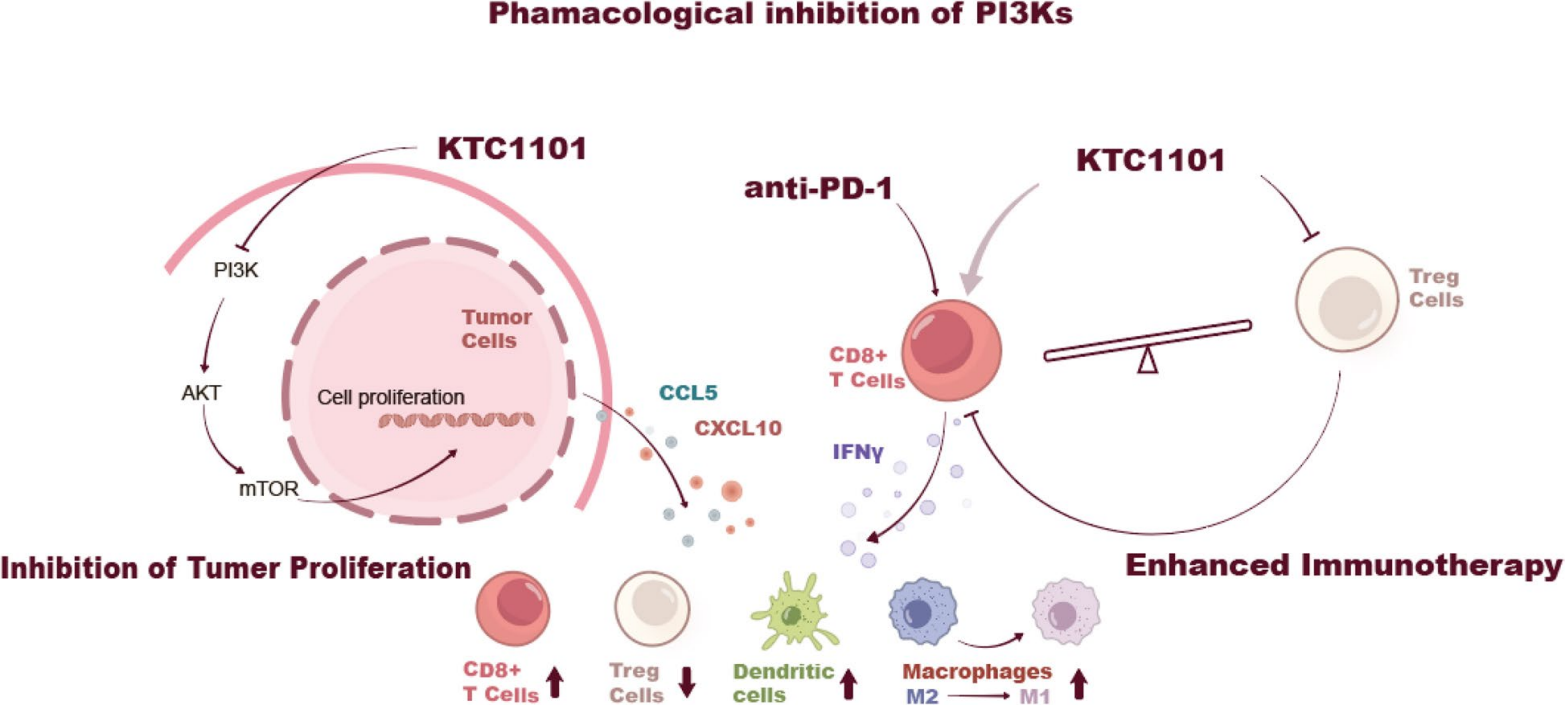
Multifaceted mechanism of KTC1101 in anti-tumor therapy and immunomodulation. The diagram delineates the inhibition of PI3K signaling within tumor cells by KTC1101, leading to reduced cell proliferation through downstream effects on AKT and mTOR pathways. Additionally, the synergistic effects of KTC1101 when combined with anti-PD-1 therapy are highlighted, showing the suppression of regulatory T cells (Tregs) and the activation of CD8^+^ T cells. This is facilitated by increased pro-inflammatory cytokines and chemokines, such as IFNγ, CCL5, and CXCL10, which promote a T-cell inflammatory environment conducive to tumor regression. The graphic also indicates the shift in macrophage phenotype from M2 to M1, signifying the immunomodulatory impact of KTC1101 on innate immune cells

Our study comprehensively unveils that KTC1101 treatment has a dual action in cancer therapy, a paradigm of the “two birds with one stone” approach. KTC1101 treatment inhibits the PI3K pathway and halts tumor growth directly, as evidenced by its capacity to induce cell cycle arrest and mitigate proliferative signaling. Concurrently, KTC1101 treatment influences the expression of tumor-derived immunomodulatory factors like CCL5 and CXCL10 that determine T-cell infiltration. Moreover, KTC1101 reduces Treg proliferation in low concentrations. KTC1101 efficacy can be enhanced by supplementation of anti-PD-1, which orchestrates a robust immune response. This is achieved by enhancing the secretion of pro-immune cytokines and chemokines, resulting in increased CD8^+^ T cell recruitment, suppression of Tregs, and an augmented infiltration of dendritic cells, along with the transition of macrophages from an M2 to an M1 phenotype within the TME (Fig. 7).

## Discussion

The development of PI3K inhibitors marks a significant stride in the landscape of cancer therapeutics. The central role of the PI3K pathway in a wide range of cellular processes positions its inhibition as a key strategic target in oncology [20, 54]. However, the journey of translating PI3K inhibitors from laboratory research to clinical settings has encountered notable challenges. These include issues related to toxicity, and mechanisms of resistance that patients may develop against these inhibitors [2, 19, 55]. Recent advancements have focused on developing inhibitors that not only target the PI3K pathway more effectively but also exhibit a better safety profile and overcome resistance issues [56, 57]. PI3K inhibitor has entailed investigations into diverse molecular structures [58], innovative dosing regimens [59], and their combinations with other therapeutic agents [21], especially within the sphere of immuno-oncology [60–63].

The interplay of negative feedback mechanisms among the four PI3K isoforms in tumor and immune cells illustrates the complexity of targeting specific isoforms for optimal therapeutic outcomes. This underscores the necessity of pursuing an optimal pan-PI3K inhibitor. Our previously developed PI3K inhibitor, ZSTK474, has already undergone clinical trials (NCT01280487, NCT01682473). In this study, we synthesized KTC1101, a novel pan-PI3K inhibitor that exhibits superior properties. KTC1101 stands out due to its favorable safety profile and pharmacokinetic properties, positioning it as a promising candidate. Notably, KTC1101 demonstrates superior efficacy compared to two significant pan-PI3K inhibitors: ZSTK474 and the only FDA-approved Copanlisib, in both in vitro and in vivo settings. This positions KTC1101 as a potentially advanced therapeutic agent in oncology, with the capability to address some of the critical challenges faced by existing PI3K inhibitors.

PI3K is known to modulate Treg activity, it also plays a vital role in activating CD8^+^ T cells. The dual role of PI3K means that its inhibition could inadvertently suppress CD8^+^ T cell activity, which is undesirable in cancer therapy where activation of anti-tumor immunity is crucial [64]. This delicate balance underscores the importance of meticulously calibrated dosing strategies. Several studies have proposed methods to activate anti-tumor immunity by designing specific dosing regimens and schedules [6, 65]. Our study revealed notable differences in the sensitivity of various T cell subsets to KTC1101, with Tregs being more susceptible compared to CD4^+^ and CD8^+^ T cells. Our comprehensive comparison of continuous and intermittent dosing regimens in syngeneic B16 mouse models, supported by RNA-Seq and flow cytometric analyses, demonstrates a distinct advantage of intermittent dosing over continuous dosing. The intermittent regimen not only effectively inhibited tumor growth but also significantly extended survival rates, likely due to its pronounced activation of the immune system. This effect is potentially linked to KTC1101's selective suppression of Tregs over CD8^+^ T cells. This beneficial outcome is potentially attributable to KTC1101's selective targeting of Tregs, while sparing CD8^+^ T cells. Our intratumoral drug concentration measurements suggest that a continuous dosing approach could disrupt this critical immunological balance, possibly leading to the unintended suppression of CD8^+^ T cells and compromising the comprehensive efficacy of the anti-tumor immune response. Therefore, our findings advocate for the strategic use of intermittent dosing as a more effective approach in leveraging the immunomodulatory potential of KTC1101 in cancer therapy.

Expanding upon these insights, we explored the synergistic potential of KTC1101 with anti-PD-1 therapy. Adjusting the sequence of administration, we found that pre-treatment with anti-PD-1 followed by combined therapy with KTC1101 yielded the most effective synergy. This approach substantially inhibited tumor growth and prolonged survival, potentially owing to increased intratumoral IFNγ post-anti-PD-1 treatment that likely renders Tregs more susceptible to KTC1101's inhibitory effects [53]. Furthermore, we noted enhanced dendritic cell infiltration and a transition from M2 to M1 macrophages in the TME. These immunological shifts, which bolster the anti-tumor response, may partly be attributed to a rise in pro-immune cytokines and chemokines in the TME. Besides, this pro-tumoral immune phenotype has also been linked to the direct targeting of specific PI3K isoforms in innate immune cells such as macrophages [11]. It is also worth noting that studies have shown isoform-selective PI3K inhibitors are less effective in suppressing Treg differentiation in vitro compared to pan-PI3K inhibitors, suggesting that pan PI3K inhibition is necessary to fully inhibit Treg differentiation [66]. This also hints at the potential superiority of pan-PI3K inhibitors. These findings highlight the imperative for ongoing research into these mechanisms, as this could lead to more refined and potent dosing strategies in future therapeutic approaches. Overall, our study demonstrates that the intermittent dosing strategy of KTC1101 in combination with anti-PD-1 therapy effectively boosts anti-tumor immunity while minimizing side effects.

As we transition these preclinical findings of KTC1101 into clinical applications, a more nuanced understanding of the intricate interplay between tumor cells and the immune system becomes crucial. Future investigations should concentrate on the enduring effects of KTC1101 on both the immune landscape and tumor control, with a specific focus on the sustainability of the immune response and the potential for resistance development. Further research will also incorporate patient-derived material, which could significantly enhance the translational value of our findings. Additionally, combining KTC1101 with other therapeutic modalities, including chemotherapy, radiation, or other forms of immunotherapy, offers a promising avenue for enhancing its overall efficacy and maximizing patient benefits.

In conclusion, KTC1101 represents a transformative advance in cancer treatment strategies, aligning with the increasing evidence supporting the use of immune-modulating agents in cancer therapy. This research provides valuable insights that could lead to potentially more effective cancer treatment strategies, particularly in the field of combining targeted therapies with immune modulation within the TME. However, further studies in more diverse and clinically relevant models are required to fully confirm the potential of KTC1101 as a promising antitumor drug candidate.

### Supplementary Information

**Supplementary Material 1.**

**Supplementary Material 2.**

**Supplementary Material 3.**

**Supplementary Material 4.**

**Supplementary Material 5.**

**Supplementary Material 6.**

**Supplementary Material 7.**

**Supplementary Material 8.**

**Supplementary Material 9.**

**Supplementary Material 10.**

**Supplementary Material 11.**

**Supplementary Material 12.**

**Supplementary Material 13.**

**Supplementary Material 14.**

**Supplementary Material 15.**

**Supplementary Material 16.**

**Supplementary Material 17.**

**Supplementary Material 18.**

**Supplementary Material 19.**

**Supplementary Material 20.**

**Supplementary Material 21.**

**Supplementary Material 22.**

**Supplementary Material 23.**

**Supplementary Material 24.**

**Supplementary Material 25.**

**Supplementary Material 26.**

## Data Availability

Data is provided within the manuscript or supplementary information files.

## Abbreviations

AKT: Protein Kinase B
ALT: Alanine Aminotransferase
AST: Aspartate Aminotransferase
BUN: Blood Urea Nitrogen
CCL5: C–C Motif Chemokine Ligand 5
CFSE: Carboxyfluorescein succinimidyl ester
CIBERSORT: Cell-type Identification by Estimating Relative Subsets of RNA Transcripts
CXCL10: C-X-C Motif Chemokine Ligand 10
ELISA: Enzyme-Linked Immunosorbent Assay
FACS: Fluorescence-Activated Cell Sorting
FL: Follicular lymphoma
GSEA: Gene Set Enrichment Analysis
GSVA: Gene Set Variation Analysis
H&E: Hematoxylin and Eosin
IFNγ: Interferon gamma
irAEs: Immune-related adverse events
LC–MS: Liquid Chromatography-Mass Spectrometry
LD50: Lethal Dose, 50%
mTOR: Mammalian Target of Rapamycin
PD-1: Programmed cell death protein 1
PI3K: Phosphoinositide 3-kinase
Rb: Retinoblastoma protein
RNA-Seq: RNA Sequencing
RT-PCR: Reverse Transcription Polymerase Chain Reaction
TME: Tumor Microenvironment
Treg cells: Regulatory T cells
