# Supplementary material for: A novel pan-PI3K inhibitor KTC1101 synergizes with anti-PD-1 therapy by targeting tumor suppression and immune activation
Source: Mol Cancer. 2024 Mar 14;23:54. doi: 10.1186/s12943-024-01978-0 (PMC10938783; doi:10.1186/s12943-024-01978-0)
Supplement: Supplementary file 1 — Supplementary Material 1. [file 12943_2024_1978_MOESM1_ESM.docx]

**Figure S1: Structural Characterization and Purity Analysis of KTC1101**

(A) ^1^H NMR Spectrum of KTC1101. (B) ^13^C NMR Spectrum of KTC1101. (C) Mass Spectrometry Analysis of KTC1101. (D) High-Performance Liquid Chromatography (HPLC) Analysis of KTC1101, verifying the compound's purity. (E) Radar chart representing KTC1101's inhibition rate across 50 kinases, with the red line indicating a 50% inhibition threshold.
