# Supplementary material for: A novel pan-PI3K inhibitor KTC1101 synergizes with anti-PD-1 therapy by targeting tumor suppression and immune activation
Source: Mol Cancer. 2024 Mar 14;23:54. doi: 10.1186/s12943-024-01978-0 (PMC10938783; doi:10.1186/s12943-024-01978-0)
Supplement: Supplementary file 2 — Supplementary Material 2. [file 12943_2024_1978_MOESM2_ESM.docx]

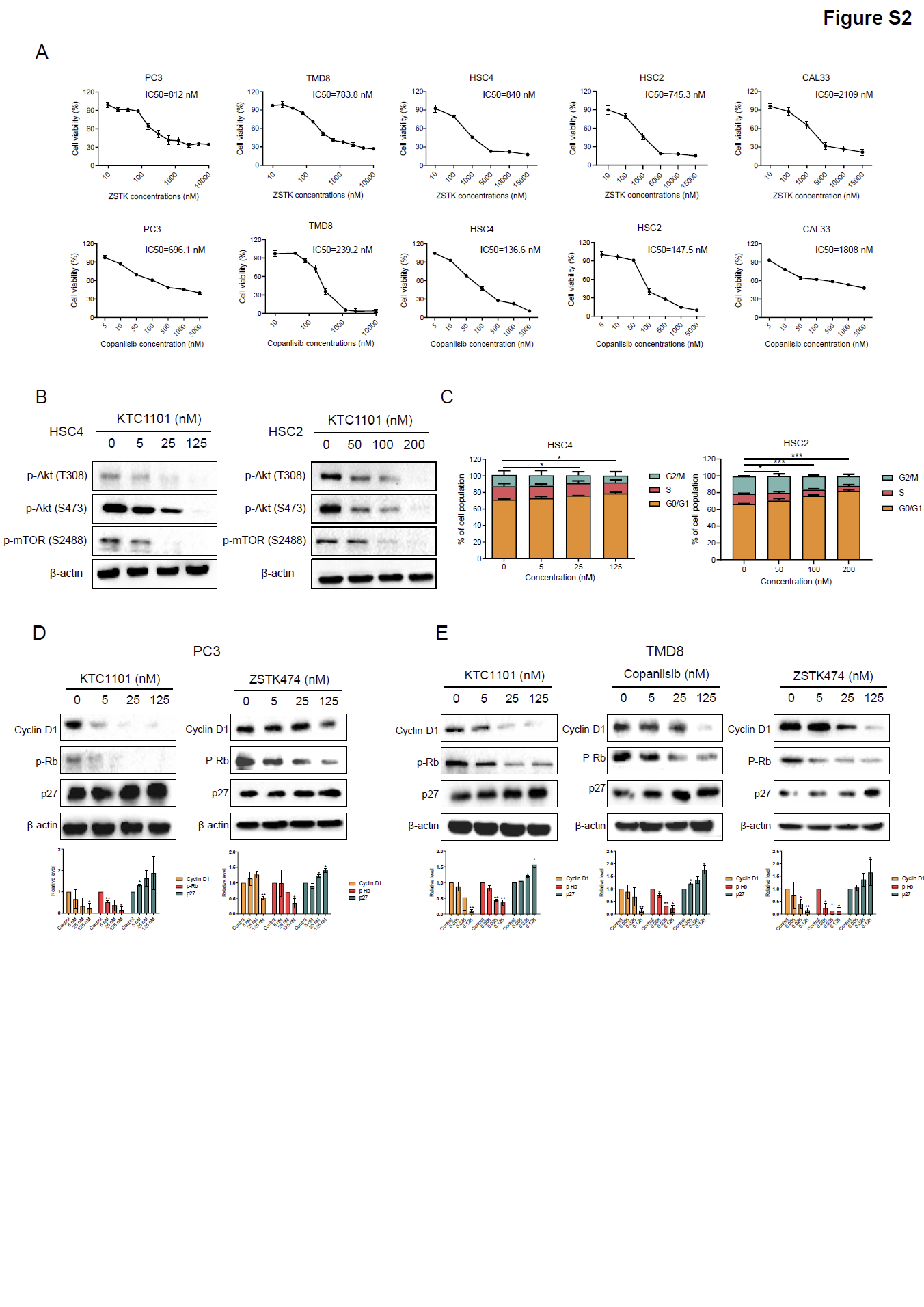


**Figure S2: Comprehensive Analysis of KTC1101's in vitro Effects**

(A) Cell viability assays illustrating KTC1101's anti-proliferative activity against various cancer cell lines after 48-hour treatment with increasing concentrations of ZSTK474 and Copanlisib. (B) Western blot analysis of Akt and mTOR phosphorylation in HSC4 and HSC2 cells after 48 hours of treatment with increasing concentrations of KTC1101. (C) Analysis of cell cycle distribution using PI staining in HSC4 and HSC2 cells after 48-hour treatment with incremental concentrations of KTC1101. (D) Analysis of cell cycle-related protein expression changes using Western blot following treatment with KTC1101 and ZSTK474 in PC3 cells for 48 hours. (E) Western blot analysis of cell cycle-related protein expression in PC3 cells, post 48-hour treatment with KTC1101, ZSTK474, and Copanlisib. Graphs are presented as the mean ± SEM from three independent experiments; P-values were determined using a two-tailed unpaired Student’s t-test; *p < 0.05; **p < 0.01; ***p < 0.001.
