# Supplementary material for: A novel pan-PI3K inhibitor KTC1101 synergizes with anti-PD-1 therapy by targeting tumor suppression and immune activation
Source: Mol Cancer. 2024 Mar 14;23:54. doi: 10.1186/s12943-024-01978-0 (PMC10938783; doi:10.1186/s12943-024-01978-0)
Supplement: Supplementary file 3 — Supplementary Material 3. [file 12943_2024_1978_MOESM3_ESM.docx]

**Figure S3: Impact of KTC1101 on Cell Apoptosis and PI3K-AKT-mTOR Signaling Pathway in vitro**

(A) Apoptosis evaluation in different cell lines treated with KTC1101 for 48 hours. (B) Quantitative assessment of apoptosis in various cell lines following KTC1101 treatment. (C) Comparison of mean GI50 values for KTC1101, ZSTK474, and Copanlisib across the JFCR39 panel. (D) Heatmap presentation of genes involved in the PI3K-AKT-mTOR signaling pathway. Graphs are presented as the mean ± SEM from three independent experiments; P-values were determined using a two-tailed unpaired Student’s t-test; **p < 0.01; ***p < 0.001.
