# Supplementary material for: A novel pan-PI3K inhibitor KTC1101 synergizes with anti-PD-1 therapy by targeting tumor suppression and immune activation
Source: Mol Cancer. 2024 Mar 14;23:54. doi: 10.1186/s12943-024-01978-0 (PMC10938783; doi:10.1186/s12943-024-01978-0)
Supplement: Supplementary file 4 — Supplementary Material 4. [file 12943_2024_1978_MOESM4_ESM.docx]

**Figure S4: In Vivo Analysis of Impacts of KTC1101**

(A) Presentation of tumors excised from nude mice with subcutaneous HSC2 tumors, treated with vehicle or various doses of KTC1101 (50 and 100 mg/kg, PO) (n = 5). (B-D) Measurements of tumor volumes, tumor weights, and body weights every three days. (E, F) NSG mice with subcutaneous tumors of CAL33 cells were treated with either a vehicle or varying doses of KTC1101 (50 or 100 mg/kg, PO). Tumor volumes and weights were measured every three days. (G) Immunohistochemistry staining of phosphorylated S6 (p-S6) and Ki67 in tumor tissues from mouse xenografts treated with KTC1101. Scale bar, 200 μm. (H) Statistical quantification of staining intensities in (G), with each point representing the mean of 5 images. (I) H&E-stained sections of major organs from treated mice. Scale bar, 200 μm. Tumor volumes Graphs are presented as the mean ± SEM from three independent experiments; P-values were determined using a two-tailed unpaired Student’s t-test; *p < 0.05; **p < 0.01; ***p < 0.001; ****p < 0.0001.
