# Supplementary material for: A novel pan-PI3K inhibitor KTC1101 synergizes with anti-PD-1 therapy by targeting tumor suppression and immune activation
Source: Mol Cancer. 2024 Mar 14;23:54. doi: 10.1186/s12943-024-01978-0 (PMC10938783; doi:10.1186/s12943-024-01978-0)
Supplement: Supplementary file 5 — Supplementary Material 5. [file 12943_2024_1978_MOESM5_ESM.docx]

**Figure S5: Cellular Response to KTC1101 in B16 Cells**

(A) Cell viability assay assessing the anti-proliferative activity of KTC1101 against B16 cells for 48 hours. (B) Western blot analysis of the phosphorylation of Akt and S6 in B16 cells treated with increasing concentrations of KTC1101 for 48 hours. (C) PI staining to determine the cell cycle distribution in B16 cells after 48-hour treatment with incremental concentrations of KTC1101. (D) Quantitative analysis of cell cycle distribution. (E) Apoptosis analysis to measure the extent of apoptosis in B16 cells treated with KTC1101 for 48 hours. (F) Quantitative analysis of apoptosis. Graphs are presented as the mean ± SEM from three independent experiments; P-values were determined using a two-tailed unpaired Student’s t-test; **p < 0.01; ***p < 0.001.
