# Supplementary material for: A novel pan-PI3K inhibitor KTC1101 synergizes with anti-PD-1 therapy by targeting tumor suppression and immune activation
Source: Mol Cancer. 2024 Mar 14;23:54. doi: 10.1186/s12943-024-01978-0 (PMC10938783; doi:10.1186/s12943-024-01978-0)
Supplement: Supplementary file 6 — Supplementary Material 6. [file 12943_2024_1978_MOESM6_ESM.docx]

**Figure S6: In Vivo Analysis of KTC1101 in B16 Models**

(A) Individual tumor growth of subcutaneous tumors of B16 cells were treated with either a vehicle or KTC1101 (n = 5). (B) Volcano plot displaying overall gene expression changes, including log fold-change values and P-values, with a horizontal dashed line indicating a P-value of 0.05. (C) Heatmap of differentially expressed genes in B16 tumor tissues following KTC1101 treatment. (D) Quantification of KTC1101 concentrations within tumor tissues in B16 models. (E) Assessment of KTC1101's anti-proliferative activity in the S24 cell line over 48 hours, conducted via cell viability assays. (F) Analysis of the phosphorylation status of Akt and S6 in the S24 cell line by Western blot after 48-hour treatment with incremental concentrations of KTC1101. (G) NSG or WT mice with subcutaneous tumors of S24 cells were treated with either a vehicle or KTC1101 (100 mg/kg, PO) daily. Tumor volumes were measured every three days. Tumor volumes Graphs are presented as the mean ± SEM from three independent experiments; P-values were determined using a two-tailed unpaired Student’s t-test; *p < 0.05.
