# Supplementary material for: A novel pan-PI3K inhibitor KTC1101 synergizes with anti-PD-1 therapy by targeting tumor suppression and immune activation
Source: Mol Cancer. 2024 Mar 14;23:54. doi: 10.1186/s12943-024-01978-0 (PMC10938783; doi:10.1186/s12943-024-01978-0)
Supplement: Supplementary file 7 — Supplementary Material 7. [file 12943_2024_1978_MOESM7_ESM.docx]

**Figure S7: Gene Expression Analysis of KTC1101's Impact on Immune-Related Signaling Pathways in B16 Cells**

(A-C) Heatmaps displaying expression patterns of genes in "IL-2-STAT5 signaling," "Inflammatory response," and "TNFα signaling via NF-κB" pathways in B16 cells treated with KTC1101 for 48 hours.
