# Supplementary material for: A novel pan-PI3K inhibitor KTC1101 synergizes with anti-PD-1 therapy by targeting tumor suppression and immune activation
Source: Mol Cancer. 2024 Mar 14;23:54. doi: 10.1186/s12943-024-01978-0 (PMC10938783; doi:10.1186/s12943-024-01978-0)
Supplement: Supplementary file 8 — Supplementary Material 8. [file 12943_2024_1978_MOESM8_ESM.docx]

**Figure S8: In Vivo Immune Modulation by KTC1101 and Anti-PD-1 Therapy**

C57BL/6J mice with subcutaneous B16 tumors were treated with either a vehicle, KTC1101, anti-PD-1 antibody or KTC1101 and anti-PD-1 antibody for 2 weeks. (A) Growth curves of individual B16 subcutaneous tumors under different treatments (n = 5). (B) Flow cytometric analysis of CD11c+ and MHCII+ DC cell populations (gated on CD45+) in B16 tumors (n = 5). (C) Flow cytometric analysis of M1 macrophage (CD86+) and M2 macrophage (CD206+) cell populations in B16 tumors (n = 5). (D) Quantification of DC cell populations in B16 tumors (n = 5). (E) Quantification of M1 macrophage cell populations in B16 tumors (n = 5). (F) Quantification of M2 macrophage cell populations in B16 tumors (n = 5). (G) Calculation of M1/M2 ratios in B16 tumors (n = 5). (H) H&E-stained sections of major organs to assess histopathological changes (scale bar: 200 μm). (I) Biochemical analysis of serum parameters including ALT, AST, BUN, and creatinine, indicating organ function. Graphs are presented as the mean ± SEM from three independent experiments; P-values were determined using a two-tailed unpaired Student’s t-test; *p < 0.05; **p < 0.01; ***p < 0.001; ****p < 0.0001.
