# Supplementary material for: A novel pan-PI3K inhibitor KTC1101 synergizes with anti-PD-1 therapy by targeting tumor suppression and immune activation
Source: Mol Cancer. 2024 Mar 14;23:54. doi: 10.1186/s12943-024-01978-0 (PMC10938783; doi:10.1186/s12943-024-01978-0)
Supplement: Supplementary file 9 — Supplementary Material 9. [file 12943_2024_1978_MOESM9_ESM.docx]

Supplementary Table 1. Primers used in this study.

| Mouse primer | 5’ to 3’ |
| --- | --- |
| mouse CCL5-F | CTCTGCCGCGGGTACCATGA |
| mouse CCL5-R | TCCTTCGAGTGACAAACACGACTGC |
| mouse CXCL10-F | GCCCACGTGTTGAGATCATTGCCA |
| mouse CXCL10-R | TGTGTGCGTGGCTTCACTCCA |
| mouse IFNγ-F | TGGAGGAACTGGCAAAAGGATGGT |
| mouse IFNγ-R | ATGCTTGGCGCTGGACCTGT |
| mouse βactin-F | CAGCCACTGTCGAGTCGCGT |
| mouse βactin-R | CACCATCACACCCTGGTGCCT |
