# Supplementary material for: A novel pan-PI3K inhibitor KTC1101 synergizes with anti-PD-1 therapy by targeting tumor suppression and immune activation
Source: Mol Cancer. 2024 Mar 14;23:54. doi: 10.1186/s12943-024-01978-0 (PMC10938783; doi:10.1186/s12943-024-01978-0)
Supplement: Supplementary file 10 — Supplementary Material 10. [file 12943_2024_1978_MOESM10_ESM.docx]

Supplementary Table 2. Antibodies used in this study.

| Name | Clone | Company | Cat # |
| --- | --- | --- | --- |
| PerCP/Cyanine5.5 anti-mouse CD3ε Antibody | 145-2C11 | Biolegend | 100328 |
| FITC anti-mouse CD4 Antibody | GK1.5 | eBioscience | 11-0041-82 |
| PE anti-mouse CD8a Antibody | 53-6.7 | eBioscience | 12-0081-82 |
| APC anti-mouse CD25 Antibody | PC61.5 | eBioscience | 17-0251-81 |
| PE anti-mouse FOXP3 Antibody | FJK-16s | eBioscience | 12-5773-82 |
| PE anti-mouse CD127 (IL-7Rα) Antibody | A7R34 | Biolegend | 135009 |
| FITC anti-mouse CD45 Antibody | 30-F11 | eBioscience | 11-0451-85 |
| APC anti-mouse CD11c Antibody | N418 | eBioscience | 17-0114-82 |
| PE anti-mouse MHC Class II (I-A/I-E) Antibody | M5/114.15.2 | eBioscience | 12-5321-82 |
| PerCP/Cyanine5.5 anti-mouse CD11b Antibody | M1/70 | eBioscience | 45-0112-82 |
| FITC anti-mouse F4/80 Antibody | BM8 | eBioscience | 11-4801-81 |
| APC anti-mouse CD86 (B7-2) Antibody | GL1 | eBioscience | 17-0862-81 |
| PE anti-mouse CD206(MMR) Antibody | MR6F3 | eBioscience | 12-2061-80 |
