# Supplementary material for: A novel pan-PI3K inhibitor KTC1101 synergizes with anti-PD-1 therapy by targeting tumor suppression and immune activation
Source: Mol Cancer. 2024 Mar 14;23:54. doi: 10.1186/s12943-024-01978-0 (PMC10938783; doi:10.1186/s12943-024-01978-0)
Supplement: Supplementary file 13 — Supplementary Material 13. [file 12943_2024_1978_MOESM13_ESM.docx]

Supplementary Table 5. Evaluation of KTC1101’s Metabolic Stability in Liver Microsomes

| Mouse Microsome Stability | *T*_1/2_ (min) | CL_int__mic (µL/min/mg protein) | CL_int__liver (mL/min/g liver) |
| --- | --- | --- | --- |
| Midazolam | 1.24 | 1115 | 4415 |
| KTC1101 | 40.7 | 34.0 | 135 |
