# Supplementary material for: A novel pan-PI3K inhibitor KTC1101 synergizes with anti-PD-1 therapy by targeting tumor suppression and immune activation
Source: Mol Cancer. 2024 Mar 14;23:54. doi: 10.1186/s12943-024-01978-0 (PMC10938783; doi:10.1186/s12943-024-01978-0)
Supplement: Supplementary file 14 — Supplementary Material 14. [file 12943_2024_1978_MOESM14_ESM.docx]

Supplementary Table 6. Assessment of KTC1101’s Metabolic Stability in Plasma

| Plasma Stability | | *T*_1/2_ (min) |
| --- | --- | --- |
| Procaine | 37.8 | |
| KTC1101 | ＞120 | |
