# Supplementary material for: A novel pan-PI3K inhibitor KTC1101 synergizes with anti-PD-1 therapy by targeting tumor suppression and immune activation
Source: Mol Cancer. 2024 Mar 14;23:54. doi: 10.1186/s12943-024-01978-0 (PMC10938783; doi:10.1186/s12943-024-01978-0)
Supplement: Supplementary file 15 — Supplementary Material 15. [file 12943_2024_1978_MOESM15_ESM.docx]

Supplementary Table 7. Pharmacokinetic Parameters of KTC1101

| Parameter | Unit (s) | 100 mg/kg p.o. |
| --- | --- | --- |
| *t*_1/2_ | h | 7.19±4.07 |
| *T*_max_ | h | 0.67±0.88 |
| *C*_max_ | μg/mL | 1.66±0.71 |
| AUC_0-t_ | μg/mL*h | 9.33±1.61 |
| Vz/F | L/kg | 117.96±85.52 |
| CLz/F | L/h/kg | 10.71±1.72 |
