# Supplementary material for: A novel pan-PI3K inhibitor KTC1101 synergizes with anti-PD-1 therapy by targeting tumor suppression and immune activation
Source: Mol Cancer. 2024 Mar 14;23:54. doi: 10.1186/s12943-024-01978-0 (PMC10938783; doi:10.1186/s12943-024-01978-0)
Supplement: Supplementary file 16 — Supplementary Material 16. [file 12943_2024_1978_MOESM16_ESM.docx]

Supplementary Table 8. Acute Toxicity Test Results for KTC1101

| Real Dose (mg/kg) | Log Dose | Score |
| --- | --- | --- |
| 1346 | 3.129 | O |
| 1750 | 3.243 | OOO |
| 2275 | 3.357 | XXX |
