# Supplementary material for: A novel pan-PI3K inhibitor KTC1101 synergizes with anti-PD-1 therapy by targeting tumor suppression and immune activation
Source: Mol Cancer. 2024 Mar 14;23:54. doi: 10.1186/s12943-024-01978-0 (PMC10938783; doi:10.1186/s12943-024-01978-0)
Supplement: Supplementary file 26 — Supplementary Material 26. [file 12943_2024_1978_MOESM26_ESM.docx]

**Supplemental Methods**

**General Procedures for Preparation of KTC1101**

**Step 1: Preparation of 4-(4,6-dichloro-1,3,5-triazin-2-yl)morpholine**

Dissolve trichloroacetonitrile (1.0 g, 5.4 mmol) in an appropriate amount of acetone. Cool the solution to -20 °C and add a solution of morpholine (0.3 g, 3.9 mmol) and triethylamine (0.4 g, 3.9 mmol) in acetone (10 mL). Stir the mixture for 30 minutes at -20 °C, monitoring the reaction progress by TLC. After completion, add water to precipitate the product, filter, wash, and dry to obtain a white solid (yield 59.7%). ^1^H NMR (400 MHz, CDCl_3_) *δ* 3.93 - 3.85 (m, 4H), 3.79 - 3.72 (m, 4H).

**Step 2: Preparation of 2-methoxy-4,6-dinitroaniline**

Dissolve 2-methoxy-4-nitroaniline (4.0 g, 24.0 mmol) in glacial acetic acid (12 mL). Slowly add concentrated H_2_SO_4_ (20 mL) to the reaction mixture at 0 °C. Add a mixture of 70% HNO_3_ (1.6 mL) and concentrated H_2_SO_4_ (1.0 mL) dropwise, then allow the reaction to proceed at room temperature, monitoring by TLC. After completion, add 100 mL of ice water to precipitate the product, filter, wash the precipitate with water, dry, and purify by column chromatography to obtain a yellow solid (yield 32.6%). ^1^H NMR (400 MHz, CDCl_3_) δ 8.74 (d, *J* = 2.4 Hz, 1H), 7.65 (d, *J* = 2.3 Hz, 1H), 3.99 (s, 3H).

**Step 3: Preparation of 3-methoxy-5-nitrobenzene-1,2-diamine**

Mix triethylamine and acetonitrile in a volume ratio of 1.2:1. Add the mixture (44 mL) to a reaction flask, along with 2-methoxy-4,6-dinitroaniline (8.0 g, 37.5 mmol) and 10% Pd/C (0.4 g). At 15 °C, add formic acid-acetonitrile solution (28 mL, volume ratio of formic acid to acetonitrile 1:2.5) and reflux for 4 hours, monitoring by TLC. Filter to remove the catalyst, concentrate under reduced pressure, and purify by column chromatography to obtain an orange solid (yield 42.2%). ^1^H NMR (400 MHz, DMSO) *δ* 7.26 (d, *J* = 2.5 Hz, 1H), 7.16 (d, *J* = 2.5 Hz, 1H), 5.61 (s, 2H), 5.13 (s, 2H), 3.83 (s, 3H).

**Step 4: Preparation of 2-(difluoromethyl)-4-methoxy-6-nitro-1*H*-benzo[*d*]imidazole**

Combine 3-methoxy-5-nitrobenzene-1,2-diamine (0.03 g, 0.2 mmol) and difluoroacetic acid (0.06 g, 0.6 mmol) in PPA (1.3 g) and react at 130°C, monitoring by TLC. After completion, add water (20 mL), adjust the pH to neutral, filter, wash the precipitate with water, and dry to obtain a dark green solid (yield 60.5%). ^1^H NMR (400 MHz, DMSO) *δ* 14.24 (s, 1H), 8.19 (s, 1H), 7.65 (s, 1H), 7.32 (t, *J* = 52.9 Hz, 1H), 4.08 (s, 3H).

**Step 5: Preparation of tert-butyl (2-(difluoromethyl)-4-methoxy-1*H*-benzo[*d*]imidazole-6-yl)carbamate**

Dissolve 2-(difluoromethyl)-4-methoxy-6-nitro-1*H*-benzo[*d*]imidazole (2.3 g, 9.5 mmol) in methanol (10 mL), add 10% Pd/C (0.1 g), and monitor the reaction by TLC. After completion, filter, concentrate the filtrate under reduced pressure. Add di-tert-butyl dicarbonate (6.4 g) and dioxane (40 mL) to the concentrated solution, reflux for 5 hours, concentrate under reduced pressure, add methanol (60 mL) and sodium hydroxide solution (2 M, 25 mL), monitored by TLC. After completion, adjust the pH to neutral, concentrate under reduced pressure, extract with ethyl acetate, and purify the organic phase by column chromatography to obtain a white solid (yield 45.9%). ^1^H NMR (400 MHz, DMSO) *δ* 13.05 (s, 1H), 9.36 (s, 1H), 7.42 (s, 1H), 7.16 (dd, *J* = 62.9, 44.0 Hz, 1H), 6.88 (s, 1H), 3.89 (s, 3H), 1.49 (s, 9H).

**Step 6: Preparation of tert-butyl (1-(4-chloro-6-morpholino-1,3,5-triazin-2-yl)-2-(difluoromethyl)-4-methoxy-1*H*-benzo[*d*]imidazol-6-yl)carbamate**

4-(4,6-Dichloro-1,3,5-triazin-2-yl)morpholine (0.5 g, 2.2 mmol), tert-Butyl (2-(difluoromethyl)-4-methoxy-1*H*-benzo[*d*]imidazole-6-yl)carbamate (0.7 g, 2.2 mmol), and potassium carbonate (K_2_CO_3_, 1.2 g, 8.6 mmol) were added to DMF (12.5 mL). The reaction was carried out at room temperature, monitored by TLC. After completion, ice water (50 mL) was added, the product was filtered, washed, and dried to yield a white solid intermediate (49.5% yield). ^1^H NMR (400 MHz, CDCl_3_) δ 8.47 (s, 1H), 7.55 (d, *J* = 21.8 Hz, 1H), 6.65 (d, *J* = 17.5 Hz, 2H), 4.17 - 4.09 (m, 2H), 4.01 (s, 3H), 3.99 - 3.94 (m, 2H), 3.92 - 3.85 (m, 2H), 3.84 - 3.77 (m, 2H), 1.52 (s, 9H).

**Step 7: Preparation of tert-butyl (2-(difluoromethyl)-4-methoxy-1-(4-morpholino-6-(1,4-oxazepan-4-yl)-1,3,5-triazin-2-yl)-1*H*-benzo[*d*]imidazol-6-yl)carbamate**

Diazepane (0.2 g, 2 mmol) and intermediate 15 (0.1 g, 0.2 mmol) were dissolved in THF (10 mL). The reaction was carried out at room temperature, monitored by TLC. After completion, water (25 mL) was added, the product was filtered, washed with methanol, and dried to yield a white solid (32.1% yield). ^1^H NMR (400 MHz, CDCl_3_) *δ* 8.61 (s, 1H), 7.54 (td, *J* = 54.0, 24.1 Hz, 1H), 6.66 (s, 1H), 6.41 (s, 1H), 4.17 - 3.63 (m, 19H), 2.04 (s, 2H), 1.51 (s, 9H).

**Step 8: Preparation of 2-(difluoromethyl)-4-methoxy-1-(4-morpholino-6-(1,4-oxazepan-4-yl)-1,3,5-triazin-2-yl)-1*H*-benzo[*d*]imidazol-6-amine (KTC1101)**

Intermediate 16 (0.1 g, 0.2 mmol) was dissolved in an appropriate amount of dichloromethane. A certain amount of trifluoroacetic acid was added, and the reaction was carried out at room temperature monitored by TLC. Upon completion, the reaction mixture was concentrated under reduced pressure, water was added, and the aqueous layer was washed with dichloromethane to remove impurities. The pH of the aqueous phase was adjusted to alkaline with potassium carbonate, washed with ethyl acetate, and the organic phase was purified by column chromatography to yield a pale yellow solid (51.9% yield). ^1^H NMR (400 MHz, DMSO) *δ* 7.62 (td, *J* = 53.5, 11.4 Hz, 1H), 7.11 (d, *J* = 13.5 Hz, 1H), 6.28 (s, 1H), 5.44 (s, 2H), 3.93 - 3.61 (m, 19H), 1.90 (dd, *J* = 11.2, 5.6 Hz, 2H). ^13^C NMR (101 MHz, DMSO) *δ* 164.51, 161.54, 151.75, 148.80, 140.87, 135.96, 123.52, 95.27, 91.36, 69.00, 68.30, 65.89, 55.34, 48.99, 45.24, 43.64, 29.24. MS (Mass Spectrometry) calcd for C_21_H_26_F_2_N_8_O_3_ [M + H]^+^: 477.2, found 477.2.
